# Dimensionality of Moral Distress Among Emergency Nurses in a Non‐Western Context: A Two‐Stage Psychometric Validation

**DOI:** 10.1155/jonm/1438300

**Published:** 2026-10-15

**Authors:** Fatemeh Monjazebi, Vahid Zamanzadeh, Fatemeh Alidadi, Fataneh Ghadirian

**Affiliations:** ^1^ Department of Medical-Surgical Nursing School of Nursing and Midwifery Shahid Beheshti University of Medical Sciences Tehran Iran sbmu.ac.ir; ^2^ Department of Management and Psychiatric Nursing School of Nursing and Midwifery Shahid Beheshti University of Medical Sciences Tehran Iran sbmu.ac.ir

**Keywords:** burnout, emergency nursing, factor analysis, measurement invariance, moral distress, professional autonomy, psychometrics, turnover intention

## Abstract

**Aim:**

To evaluate the dimensional structure of moral distress in a non‐Western emergency nursing context, cross‐validate it in an independent subsample, and establish concurrent criterion‐related validity against burnout and turnover intention.

**Background:**

Moral distress predicts burnout and turnover in emergency nursing but is usually analyzed as a single aggregate score, treating structurally distinct ethical conflicts as interchangeable.

**Methods:**

Two‐stage split‐half psychometric study across six university‐affiliated emergency departments in western Iran, reported per COSMIN and STROBE. Of 620 eligible nurses, 568 consented (91.6%) and were randomly allocated to exploratory (*n* = 298) and confirmatory (*n* = 242) subsamples after listwise deletion (pooled *N* = 540). Participants completed the culturally adapted 38‐item Moral Distress Scale, the Maslach Burnout Inventory, and a turnover intention measure. Both subscales were analyzed by principal axis factoring with parallel analysis; the intensity model was tested by confirmatory factor analysis, compared against a unidimensional alternative, and examined for configural invariance. Hierarchical regression quantified incremental variance in the outcomes.

**Results:**

Exploratory analysis yielded congruent five‐factor solutions across both subscales (mean Tucker’s *φ* = 0.988 and Kaiser–Meyer–Olkin = 0.955 and 0.943): communication and autonomy conflicts, unprofessional and futile care, systemic and economic constraints, working with incompetent colleagues, and conflicts with family wishes. All 38 items loaded on their expected factor (0.777–0.899) with no cross‐loading above 0.111. Confirmatory fit was excellent (*χ* ^2^[655] = 731.00; comparative fit index [CFI] = 0.991; root mean square error of approximation [RMSEA] = 0.022; and standardized root mean square residual = 0.034) and held separately in educational and noneducational hospitals. Reliability (*α* = 0.925–0.963) and convergent validity (average variance extracted = 0.689–0.774) were supported. The dimensions added 49.4%, 40.5%, and 46.6% of variance in emotional exhaustion, depersonalization, and turnover intention (all *p* < 0.001), with communication and autonomy conflicts the strongest correlate throughout (*β* = 0.274–0.341). A unidimensional model fitted poorly (CFI = 0.415 and RMSEA = 0.176).

**Conclusions:**

Moral distress was better represented as multidimensional than unidimensional. Constrained professional voice was the dimension most strongly associated with workforce vulnerability, identifying it as a priority for organizational attention. Associations are concurrent, invariance was established only at the configural level, and cross‐national replication is required.

**Implications for Nursing Management:**

Nurse managers should move beyond aggregate moral distress scores and identify the specific ethical conflict domains that place emergency nurses at greatest risk. Management strategies should prioritize strengthening nurses’ professional voice, improving interdisciplinary communication, supporting ethical decision‐making, and addressing organizational constraints that limit autonomy. Targeted interventions in these areas may help reduce burnout, improve retention, and create ethically supportive emergency care environments.

## 1. Introduction

The emergency department (ED) operates as a high‐velocity, complex adaptive system in which clinical decisions must be executed rapidly under conditions of diagnostic uncertainty, resource scarcity, and emotional volatility [1, 2]. Several structural features distinguish this environment from other acute care settings: patients present undifferentiated and often without accessible histories; decisions are compressed into short timeframes; nurses rarely have a prior therapeutic relationship with the patient or family; and admitted patients may be boarded for extended periods in an environment not designed for continuing care. Each of these features plausibly increases the frequency with which emergency nurses encounter situations in which the ethically appropriate action is identifiable but obstructed, and it is this concentration of exposure within the ED setting—rather than any indicative relationship to conditions elsewhere in the hospital—that motivates the present focus.

The governing theoretical framework for this study is Jameton’s [3] formulation of moral distress, as elaborated in subsequent theoretical work. Jameton distinguished moral distress from moral uncertainty and moral dilemma: distress arises specifically when a practitioner recognizes the ethically appropriate course of action but is prevented from pursuing it by an external constraint. Three features of this formulation are consequential for measurement. First, the construct presupposes moral judgment, so it cannot be reduced to generalized occupational stress. Second, it locates causation in constraint rather than in individual deficiency, which directs attention to organizational conditions. Third, and most importantly for the present study, the constraints are heterogeneous in kind—Jameton and subsequent authors identify institutional policy, hierarchical authority, resource limitation, and interpersonal conflict as distinct sources [4, 5].

Epstein and Hamric [6] extended this framework through the concepts of moral residue and the crescendo effect, proposing that unresolved episodes leave a durable deposit that accumulates across a career so that each subsequent episode is experienced against a rising baseline. Morley et al. [5], in a narrative synthesis, argued for a broader definition encompassing distress arising from moral uncertainty and from the experience of moral constraint irrespective of whether a clear ethical judgment preceded it. We adopt the constraint‐centered formulation while acknowledging this contested boundary.

It is the third feature—the heterogeneity of constraints—that generates the measurement problem addressed here. If moral distress arises from structurally distinct sources, then a single aggregate score imposes a strong and rarely examined assumption: that a nurse silenced by a physician’s disregard and a nurse unable to obtain essential supplies are experiencing the same quantity of the same construct. Nothing in the underlying theory supports this equivalence. Aggregation may, therefore, obscure the etiology of distress and offer little guidance to managers, for whom the appropriate response to hierarchical silencing differs substantially from the appropriate response to supply shortage [7]. Deconstructing the construct into its constituent dimensions is thus a prerequisite for identifying which conflicts are most closely associated with workforce attrition, not merely a psychometric refinement.

A substantial literature links moral distress with emotional exhaustion, depersonalization, compassion fatigue, and turnover‐related outcomes among emergency and critical care nurses [2, 8–10]. A predictive study of 290 emergency nurses in Saudi Arabia found that sociodemographic and occupational factors accounted for 67.9% of the variance in burnout, compassion fatigue, and moral distress, with night‐shift patterns and educational attainment emerging as salient markers [8]. Recent work also positions moral distress on a continuum toward moral injury: a review of medical personnel and first responders reported prevalence estimates from 4.1% to 69.4% across settings and identified organizational constraint, traumatic exposure, and absence of support as recurrent antecedents [11].

Two gaps remain. The first concerns validity evidence. Psychometric work on moral distress frequently terminates at internal structure, establishing that a factor solution fits without demonstrating that the resulting dimensions relate to outcomes of practical consequence [12]. Dimensional models that are statistically defensible but unconnected to burnout or attrition offer managers little actionable information. The second concerns cultural transportability. Most validated structures originate in Western, individualistic healthcare frameworks in which patient autonomy is the organizing ethical principle. In the Iranian system, steeper physician–nurse authority gradients, family‐centered decision‐making grounded in Islamic bioethical reasoning, and macroeconomic constraint on supply availability together constitute a different moral landscape [13, 14]. A recent psychometric evaluation of the Persian Moral Distress Scale–Revised among pediatric nurses in Tehran identified four factors—limited provider competency, care under multifactorial pressure, futile care, and lack of moral courage—a structure that does not straightforwardly reproduce Western solutions and underscores the need for setting‐specific dimensional models [15]. Examining dimensionality in this context, therefore, functions as a test of whether existing structures hold where the surrounding ethical architecture differs.

Against this background, the present study pursued five objectives:

1. To cross‐culturally adapt Corley’s Moral Distress Scale for Iranian emergency nurses and evaluate its measurement properties;
2. To identify the latent dimensional structure of both frequency and intensity subscales through exploratory factor analysis;
3. To cross‐validate the intensity‐based structure through confirmatory factor analysis in an independent split‐half subsample;
4. To establish concurrent criterion‐related validity by examining associations between dimensions and burnout and turnover intention; and
5. To compare the validated dimensions by standardized intensity.

## 2. Materials and Methods

### 2.1. Study Design and Setting

A two‐stage descriptive psychometric study with concurrent criterion‐related validation was conducted from 1 June to 30 September 2025 across six university‐affiliated public hospitals in three geographically and demographically distinct cities in western Iran. Each city is served by an independent University of Medical Sciences: City A, a large metropolitan area (population≈850,000); City B, a medium‐sized city (≈420,000); and City C, a small city (≈180,000). Within each city, one educational and one noneducational public hospital were purposively selected, yielding six EDs across three university jurisdictions. This stratification ensured variation in urban density, patient acuity, referral pattern, and organizational culture while maintaining public‐sector comparability. Social security, private, and military hospitals were excluded. City and university names are withheld to protect institutional anonymity. The study was designed and reported in accordance with COSMIN principles for studies of measurement properties and the STROBE statement [16, 17].

### 2.2. Participants and Sampling

The target population comprised all registered nurses providing direct patient care in the six selected EDs during the study period. Total‐population (census) sampling was employed at the participant level: every eligible nurse on active clinical duty across all six departments and all shift rotations was invited, with no sampling fraction applied and no convenience or quota selection. Purposive selection operated only at the institutional level, in the choice of the six hospitals described above.

Inclusion criteria were active nursing licensure with at least a baccalaureate degree; a minimum of 6 months of ED clinical experience; direct involvement in bedside care during data collection; and written informed consent. Exclusion criteria were participation in formal ethics or moral resilience training within the preceding 6 months; current assignment to administrative, educational, or other nonclinical roles; and self‐reported active treatment for a psychiatric condition that could confound psychological assessment.

Of 620 eligible nurses, 568 consented and completed questionnaires, yielding a 91.6% response rate. Anonymized demographic data were available for 48 of the 52 nonrespondents. Respondents and nonrespondents were compared using chi‐square tests, independent‐samples *t*‐tests, and Two One‐Sided Tests for equivalence with an equivalence bound of Cohen’s *d* = ±0.25 [18, 19]; no meaningful demographic differences were detected.

Of the 568 consenting participants, cases were randomly allocated to an EFA subsample (*n* = 312) and an independent CFA subsample (*n* = 256) using a computer‐generated random sequence. Cases with any missing item response were removed by listwise deletion. In the EFA subsample, 14 cases were deleted (11 with incomplete Moral Distress Scale responses, 2 with incomplete Maslach Burnout Inventory responses, and 1 with an incomplete demographic form), yielding *n* = 298. In the CFA subsample, 14 cases were deleted (10 incomplete Moral Distress Scale, 3 incomplete Maslach Burnout Inventory, and 1 incomplete Turnover Intention Scale), yielding *n* = 242. The pooled analytic sample was, therefore, 540, representing 95.1% of consenting participants and 87.1% of the 620 eligible nurses. No missing values occurred on any Moral Distress Scale item. All deletions arose from incomplete Maslach Burnout Inventory responses (151 missing cells across the 28 cases, 2–8 per case), 26 of which were also incomplete on the turnover measure. Deleted and retained cases did not differ on any measured demographic or occupational characteristic (all *p* > 0.36), supporting the assumption that the deletions were unrelated to the constructs under study. Participant flow is summarized in Figure 1.

**FIGURE 1:**
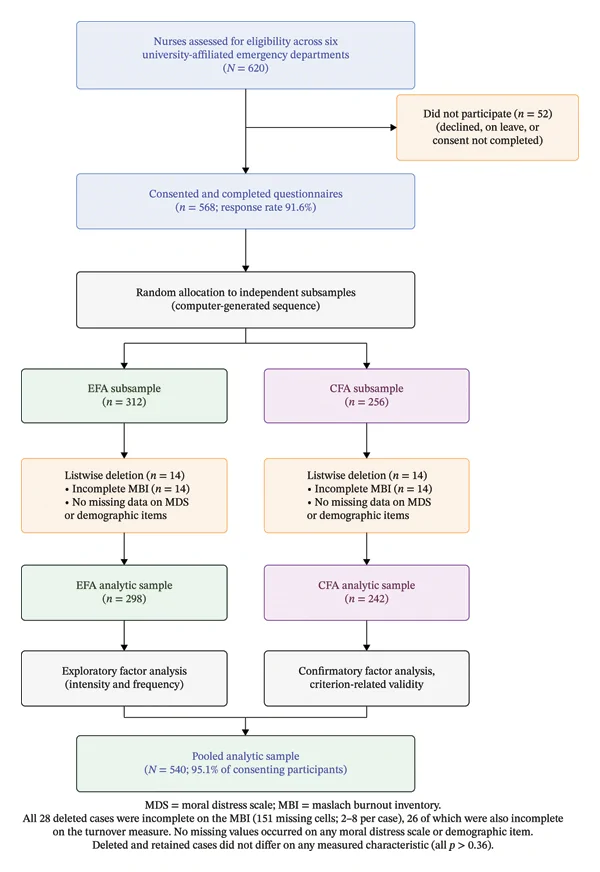
Participant flow through eligibility, consent, random allocation, and listwise deletion to the final analytic samples.

The two subsamples were equivalent on all demographic and occupational variables under both conventional significance testing and TOST equivalence testing with *d* = ±0.20. For the exploratory stage, the respondent‐to‐item ratio was 7.8:1 (298/38), exceeding the conventional minimum of 5:1. For the confirmatory stage, *n* = 242 exceeded the commonly recommended minimum of 200 for models of this complexity [20].

### 2.3. Ethical Considerations

The protocol was approved by the Research Ethics Committee of Shahid Beheshti University of Medical Sciences (IR.SBMU.PHARMACY.REC.1403.052). Official permissions were obtained from hospital administrators and ED chiefs. Written informed consent was obtained from all participants following explanation of the objectives, confidentiality procedures, and the right to withdraw without consequence. Completed questionnaires were deposited in sealed, nonidentifiable envelopes. No financial incentives were offered.

### 2.4. Instruments

Demographic and professional characteristics form.

A researcher‐designed form recorded age, gender, marital status, educational attainment, total nursing experience, ED‐specific experience, employment status, shift type, weekly working hours, and hospital type.

Moral Distress Scale: The Moral Distress Scale was developed by Corley et al. [21] and subsequently used in an expanded 38‐item form in research on nurses’ moral distress and ethical work environment [22]. The 38‐item version was selected because it retains paired frequency and intensity ratings and provides a broader item pool for exploratory dimensional analysis in the ED context. Written permission to use the Moral Distress Scale was granted by Professor Mary C. Corley, the lead developer of the instrument. The permission email is provided as Supporting File A. Adaptation followed established cross‐cultural guidance [23, 24] with full attribution to the original developers. Each situation is rated on two 7‐point scales: frequency from 0 (*never*) to 6 (*very frequently*) and intensity from 0 (*none*) to 6 (*to a great extent*). All 38 items were retained. Abbreviated item content is given in Supporting Tables S1 and S2; the full validated wording is not reproduced here, in keeping with the copyright status of the instrument. Persian adaptations of related instruments exist—the Moral Distress Scale—Revised has been validated in Iranian nurses [25, 26] and a 15‐item psychiatric version has been developed [27]—but no validated Persian version of the full 38‐item instrument was available, and its dimensional structure had not been examined in an Iranian emergency nursing population. This provided the rationale for the present adaptation and psychometric evaluation.

The intensity subscale was specified as the primary model for confirmatory analysis and criterion‐related validation for four reasons. Theoretically, moral distress is defined by the severity of psychological disequilibrium rather than by the frequency of precipitating events, making intensity the closer operationalization of the construct. Psychometrically, frequency ratings function as retrospective event counts subject to recall bias and are strongly influenced by unit case‐mix, introducing setting‐attributable variance. Substantively, the criterion outcomes are affective and attitudinal states for which experienced severity is the conceptually appropriate correlate. Empirically, the near‐identity of the two exploratory solutions (mean Tucker’s *φ* = 0.988; see Supporting Table S3, Panel C) indicated that a separate confirmatory model for frequency would test an essentially equivalent structure.

No validated diagnostic cutoff exists for the Moral Distress Scale, and none is proposed here. Establishing a defensible threshold requires calibration against an external criterion—a diagnostic interview or a prospective outcome such as actual resignation—and no such anchor was collected. Where proportions scoring above the scale midpoint are reported below, the midpoint serves as an arbitrary descriptive reference point and carries no clinical or diagnostic meaning. Maslach Burnout Inventory–Human Services Survey.

Burnout was measured with the 22‐item Maslach Burnout Inventory–Human Services Survey [28], comprising Emotional Exhaustion, Depersonalization, and Personal Accomplishment subscales. The Persian version has been validated in Iranian nurses, with the three‐factor structure supported and acceptable internal consistency reported [29, 30]. Items are rated from 0 (*never*) to 6 (*every day*). Internal consistency in this sample was *α* = 0.908 for Emotional Exhaustion, *α* = 0.852 for Depersonalization, and *α* = 0.881 for Personal Accomplishment. Conventional descriptive bands are used to characterize the distribution; these are normative reference categories rather than diagnostic thresholds.

Turnover Intention Scale: Turnover intention was assessed with three items measuring intention to leave the organization within the next 12 months, rated from 1 (*strongly disagree*) to 5 (*strongly agree*). The item content derives from Mobley et al. [31], whose study examined precursors of turnover among hospital employees across occupational groups, of which nursing staff formed one component. Because that early work provides limited psychometric evidence, contemporary support is drawn from Bothma and Roodt [32], who validated the Turnover Intention Scale in a census‐based sample of 2429 employees, demonstrating internal consistency (*α* = 0.80) and criterion‐predictive validity against actual turnover by discriminating leavers from stayers, and from Lee et al. [9], who applied a comparable measure in emergency nurses. The three items were translated for this study following the same forward–backward procedure used for the Moral Distress Scale; we are not aware of a previously validated Persian version of this brief measure, which should be borne in mind when interpreting its performance. In the present sample, internal consistency was acceptable (*α* = 0.767); this value, while above the conventional 0.70 threshold, is modest and reflects the brevity of the three‐item measure.

### 2.5. Cross‐Cultural Adaptation

Translation and cultural adaptation followed Beaton et al. [23] and ISPOR principles for the translation and cultural adaptation of patient‐reported outcome measures [24], implemented in six stages. In stage one, two independent bilingual translators, one with a clinical nursing background and one without, produced Persian forward translations. In stage two, a reconciled version was agreed by consensus with a third bilingual reviewer adjudicating discrepancies. In stage three, two independent native English speakers blind to the source instrument backtranslated the reconciled Persian version. In stage four, an expert committee comprising two nursing ethicists, two senior ED clinicians, one psychometrician, one linguist, and one legal medicine specialist compared all versions and resolved semantic, idiomatic, experiential, and conceptual equivalence. In stage five, face validity was assessed through cognitive interviewing with 20 ED nurses using concurrent think‐aloud and verbal probing [33]. In stage six, the prefinal version underwent pilot testing.

Cultural adaptation extended beyond linguistic equivalence, and the substantive decisions are documented in Table 1. Three classes of item required attention. Items presupposing individual patient autonomy as the default ethical standard were reworded to accommodate the family as a legitimate participant in decision‐making, without presupposing either individual or family primacy, since the source phrasing framed family involvement as inherently an infringement.

**TABLE 1:** Cultural adaptation decisions applied during expert committee review.

Source item domain	Cultural issue identified	Adaptation adopted
Items presupposing individual patient autonomy	Source phrasing treats family participation in decisions as inherently an infringement of autonomy; in Iranian practice, family involvement is normative and legally recognized	Reworded to admit the family as a legitimate participant without presupposing either individual or family primacy so that distress is attributed to conflict rather than to family involvement per se
Items referring to futile care	Literal Persian rendering of “futile” connotes abandonment, conflicting with Islamic obligations of continued care and likely to provoke response bias	Rephrased to convey nonbeneficial or disproportionate treatment while explicitly preserving the obligation of comfort and supportive care
Items concerning physician orders	Source wording implies a professional latitude to decline orders that does not correspond to the steeper authority gradient of local practice	Calibrated to reflect realistic local latitude, focusing on the experience of constraint rather than on a presumed capacity to refuse
Items concerning disclosure of information	Family‐mediated disclosure is common practice; direct disclosure to the patient is not the invariable default	Reframed around conflict between the nurse’s judgment and the disclosure arrangement, rather than assuming nondisclosure is itself the ethical breach
Items concerning resource limitation	Economic constraint in this system arises substantially from macroeconomic conditions affecting supply availability rather than from insurance‐driven rationing	Wording generalized to encompass supply unavailability and cost pressure without presupposing an insurance‐based mechanism

*Note:* Adaptation followed Beaton et al. [23] and Wild et al. [24]. All decisions were agreed by the expert committee and subsequently tested through cognitive interviewing with 20 emergency nurses.

Items concerning futile care required care because the literal Persian rendering of “futile” carries connotations of abandonment that conflict with Islamic obligations of continued care; phrasing was adjusted to convey nonbeneficial or disproportionate treatment while preserving the obligation of comfort care. Items concerning physician orders were calibrated to the steeper authority gradient of Iranian practice, where the source wording implied a latitude to refuse that does not reliably correspond to local professional reality.

Cognitive interviewing led to minor lexical refinement of three items and identified MDS‐37 and MDS‐38 as the items most frequently queried by participants, who reported difficulty because each combined two distinct referents; this observation is revisited in Section 3.4. Content validity was assessed by 10 experts, with I‐CVI exceeding 0.79 for all items and S‐CVI/Ave of 0.94. Pilot testing with 30 ED nurses not included in the main study indicated excellent internal consistency (*α* = 0.92 and *ω* = 0.93) and acceptable two‐week test–retest reliability (ICC = 0.84, 95% CI [0.71, 0.92]).

### 2.6. Data Analysis

Analyses were conducted in SPSS 26, jamovi 2.7, and R 4.5 (lavaan). Hospital‐level and city‐level intraclass correlations for total moral distress were inspected descriptively. Given the small number of clusters, multilevel modeling was not feasible; single‐level models were, therefore, estimated and the hospital type was entered as a covariate in regression analyses.

Item analysis preceded structural analysis. For each item, we computed the mean and standard deviation, the difficulty index, skewness and kurtosis, the corrected item–total correlation, Cronbach’s alpha if the item were deleted, and the range of interitem correlations. Items were flagged for review where the corrected item–total correlation fell below 0.30, where deletion would have increased total alpha, or where any interitem correlation exceeded 0.80.

Exploratory factor analysis was conducted separately on the intensity and frequency subscales using Principal Axis Factoring with Promax rotation. Principal Axis Factoring was selected over Principal Components Analysis because the objective was to identify latent constructs underlying shared item variance rather than to reduce data dimensionality; Promax rotation was selected because the dimensions were expected to correlate. Factor retention was determined by convergence across three criteria: scree plot inspection, eigenvalues greater than one, and parallel analysis with 1000 permutations [34, 35], with the 95th percentile of random eigenvalues as the retention threshold. Items were retained where the primary loading was ≥ 0.40 and no cross‐loading reached 0.30. Congruence between the intensity and frequency solutions was assessed using Tucker’s congruence coefficient, with values ≥ 0.90 interpreted as evidence of factorial similarity [36].^1^

The five‐factor intensity model was tested in the independent CFA subsample using maximum likelihood estimation. Fit was evaluated against CFI and TLI ≥ 0.90, RMSEA ≤ 0.08, and SRMR ≤ 0.08 [37]. Convergent validity was assessed through average variance extracted and composite reliability, with AVE ≥ 0.50 and CR ≥ 0.70 as criteria [38]. Discriminant validity was assessed by two independent criteria: the heterotrait–monotrait ratio, with values below 0.85 taken as supportive [39] and the Fornell–Larcker criterion, requiring the square root of AVE for each dimension to exceed its correlations with all other dimensions.

Concurrent criterion‐related validity was assessed through hierarchical regression predicting emotional exhaustion, depersonalization, and turnover intention, with demographic and occupational covariates entered at step one and the five dimension scores entered simultaneously at step two. Dimension rankings were compared using the Friedman test with Kendall’s W as the effect size. A unidimensional model, specifying all 38 items as indicators of a single latent factor, was estimated to determine whether the dimensions could defensibly be collapsed. Configural invariance across the hospital type was examined by estimating the model separately within each group and comparing fit, loading patterns, and interfactor correlations. Metric and scalar invariance were not tested; the rationale and implications are addressed in Sections 3.5 and 4.4.

## 3. Results

### 3.1. Sample Characteristics

Of 620 eligible nurses, 568 consented and were randomly allocated to the two subsamples; after listwise deletion the analytic samples comprised 298 (EFA) and 242 (CFA) nurses, giving a pooled analytic sample of 540. Participant flow is shown in Figure 1. The pooled sample was predominantly experienced, married, and working rotating shifts; characteristics are presented in Table 2.

**TABLE 2:** Demographic and professional characteristics of the pooled analytic sample.

Characteristic	Category	*n*	%
Gender	Female	323	60
	Male	217	40

Age group (years)	20–29	146	27
	30–39	280	52
	≥ 40	114	21

Marital status	Married	323	60
	Single, divorced, or widowed	217	40

Education	BSc	501	93
	MSc	39	7

Hospital type	Educational	275	51
	Noneducational	265	49

Total nursing experience	< 1 year	46	9
	2–5 years	64	12
	> 5 years	430	80

ED experience (years)	< 1	38	7
	1–3	85	16
	> 3	417	77

Employment	Permanent	437	81
	Contractual	103	19

Shift type	Fixed	191	35
	Rotating	349	65

*Note: N* = 540 for every characteristic; percentages are column percentages of 540 and are rounded to whole numbers, so columns may not sum to 100. Continuous variables: age *M* = 33.97 years (SD = 6.69); emergency department (ED) experience *M* = 5.43 years (SD = 2.98). Of the 568 nurses who consented, 28 (4.9%) had at least one missing item on the Maslach Burnout Inventory or the turnover intention scale (151 of 12,496 burnout item responses, 1.2%; 26 of 1704 turnover item responses, 1.5%) and were removed by listwise deletion; no moral distress item and no demographic field was missing for any participant.

### 3.2. Descriptive Findings

Mean total moral distress intensity was 127.72 (SD = 42.69), with 61.5% of participants scoring above the scale midpoint of 114; mean frequency was 90.40 (SD = 32.44), with 25.4% above the midpoint. As noted in Section 2.4, the midpoint is an arbitrary descriptive reference and does not denote a clinically meaningful threshold. Intensity ratings exceeded frequency ratings across all five dimensions (ratio of frequency to intensity = 0.69–0.73), indicating that morally distressing situations were experienced as more severe than they were commonly encountered. Mean emotional exhaustion was 31.08 (SD = 10.45), mean depersonalization was 12.78 (SD = 5.76), and mean personal accomplishment was 26.91 (SD = 8.63). Mean turnover intention was 9.33 (SD = 2.90) on a 3–15 scale. Item‐level skewness (−0.39 to 0.23) and kurtosis (−1.25 to −0.67) fell within conventional bounds for univariate normality. These distributions indicate elevated moral distress and burnout indicators in this sample, though in the absence of validated thresholds they should be read as descriptive rather than diagnostic.

### 3.3. Item Analysis

Item‐level statistics for all 38 items are reported in Supporting Table S1. Corrected item–total correlations ranged from 0.539 to 0.639 (*M* = 0.588), with every item exceeding not only the 0.30 criterion but also the more stringent 0.50 threshold. Item difficulty indices ranged from 0.44 to 0.61, indicating adequate distributional spread without floor or ceiling effects. Cronbach’s alpha if item deleted ranged from 0.954 to 0.955 against a total‐scale alpha of 0.956, so no item deletion would have improved internal consistency. Items MDS‐37 and MDS‐38 recorded the lowest item means (2.68 and 2.64 against a range of 3.15–3.63 for the remaining items), consistent with their content addressing less frequently encountered situations, but their item–total correlations (0.549 and 0.540) were unremarkable.

### 3.4. Factor Structure

Both subscales were highly suitable for factor analysis. For intensity, KMO was 0.955, indicating marvelous sampling adequacy, and Bartlett’s test of sphericity was significant, *χ* ^2^(703) = 10507.47, *p* < 0.001. For frequency, KMO was 0.943 and Bartlett’s test was significant, *χ* ^2^(703) = 7659.67, *p* < 0.001.

Parallel analysis, scree inspection, and the eigenvalue criterion converged unambiguously on a five‐factor solution for both subscales (Figure 2). For intensity, the first five eigenvalues (14.198, 5.950, 4.149, 2.443, and 2.076) each exceeded the corresponding 95th percentile of eigenvalues from 1000 random datasets (1.842, 1.728, 1.649, 1.581, and 1.527), whereas the sixth (0.508) fell far below its random counterpart (1.475). The frequency subscale reproduced this pattern (eigenvalues 11.407, 5.598, 4.339, 2.351, and 1.827, followed by 0.666). The five‐factor solution accounted for 72.30% of extracted common variance for intensity and 62.26% for frequency. The complete rotated pattern matrix, including all cross‐loadings and communalities, is provided in Supporting Table S2, and factor correlations, eigenvalues, and congruence coefficients in Supporting Table S3.

**FIGURE 2:**
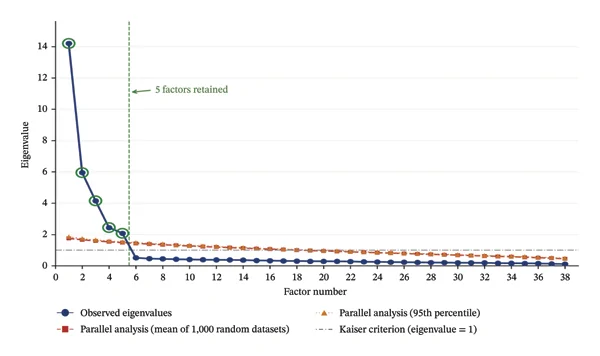
Scree plot with parallel analysis for the moral distress scale intensity subscale (EFA subsample, *n* = 298).

The five retained dimensions were (1) Communication and Autonomy Conflicts; (2) Unprofessional and Futile Care; (3) Systemic and Economic Constraints; (4) Working with Incompetent Colleagues; and (5) Conflicts with Family Wishes. The solution exhibited exemplary simple structure. Every one of the 38 items loaded on its theoretically expected factor, with primary pattern coefficients ranging from 0.777 to 0.899 for intensity and 0.692 to 0.847 for frequency. No item produced a cross‐loading of 0.30 or above in either solution; the largest absolute nonprimary coefficient was 0.111 for intensity and 0.113 for frequency. Communalities ranged from 0.617 to 0.803 (*M* = 0.723) for intensity and 0.522 to 0.706 (*M* = 0.621) for frequency. The two solutions were near‐identical in structure, with Tucker’s congruence coefficients of 0.993 (F1), 0.987 (F2), 0.988 (F3), 0.976 (F4), and 0.995 (F5), yielding a mean of 0.988. All values exceeded the 0.95 criterion for factorial equivalence [36], indicating that the dimensional architecture is essentially invariant across the two phenomenological facets of moral distress.

Particular attention was given to MDS‐37 and MDS‐38, the two items flagged during cognitive interviewing as potentially double‐barreled. Both performed adequately in the confirmatory model, with standardized loadings of 0.732 and 0.741. Although these were the lowest values in the instrument, both comfortably exceeded conventional retention thresholds. Both items were, therefore, retained, and the instrument is reported throughout in its full 38‐item form.

The five‐factor intensity model showed excellent fit in the independent CFA subsample, estimated by maximum likelihood in lavaan: *χ* ^2^(655) = 731.00, *p* = 0.021; *χ* ^2^/df = 1.12; CFI = 0.991; TLI = 0.990; RMSEA = 0.022 [0.009, 0.030]; and SRMR = 0.034. All 38 standardized loadings were substantial and significant (*λ* = 0.732–0.910, all *p* < 0.001), and modification indices identified no substantively interpretable misspecification, the largest residual covariance index being 9.91. The measurement model is displayed in Figure 3, and item‐level loadings with model comparison statistics are given in Supporting Table S4.

**FIGURE 3:**
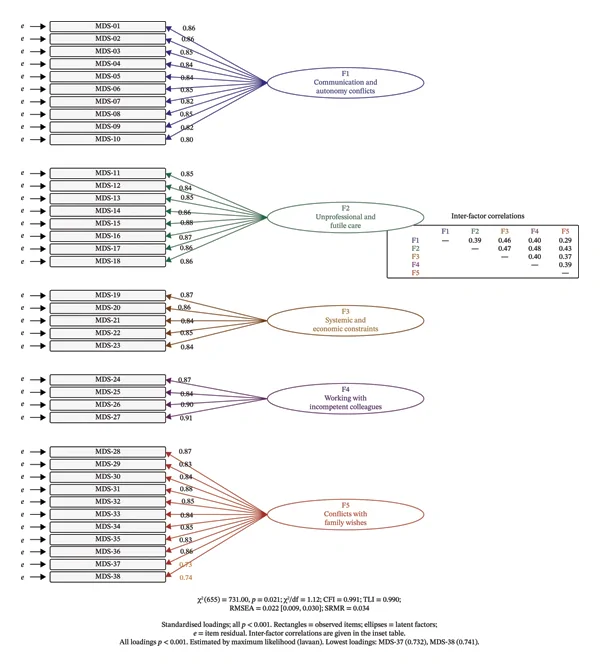
Confirmatory factor analysis measurement model of the five‐factor moral distress intensity model (CFA subsample, *n* = 242). *χ* ^2^(655) = 731.00, *p* = 0.021; CFI = 0.991; TLI = 0.990; RMSEA = 0.022 [0.009, 0.030]; and SRMR = 0.034).

Internal consistency was excellent for every dimension: Cronbach’s *α* was 0.958 for Communication and Autonomy Conflicts, 0.955 for Unprofessional and Futile Care, 0.941 for Systemic and Economic Constraints, 0.925 for Working with Incompetent Colleagues, and 0.963 for Conflicts with Family Wishes, with a total‐scale *α* of 0.956. Convergent validity was strongly supported. average variance extracted ranged from 0.689 to 0.774 and composite reliability from 0.930 to 0.960, with every dimension exceeding the 0.50 and 0.70 criteria by a substantial margin [38]. Discriminant validity was supported: the maximum interfactor correlation was 0.482, and the Fornell–Larcker criterion was satisfied for every dimension, with the square root of AVE (0.830–0.880) exceeding all corresponding inter‐factor correlations. Full values are reported in Table 3 and Supporting Table S5.

**TABLE 3:** Internal consistency, convergent validity, and discriminant validity of the five moral distress dimensions.

Dimension	*k*	*α*	95% CI for *α*	CR	AVE	√AVE	Max *r*
F1 Communication and Autonomy Conflicts	10	0.958	0.952, 0.963	0.960	0.704	0.839	0.456
F2 Unprofessional and Futile Care	8	0.955	0.948, 0.961	0.958	0.741	0.861	0.482
F3 Systemic and Economic Constraints	5	0.941	0.932, 0.948	0.930	0.728	0.853	0.474
F4 Working with Incompetent Colleagues	4	0.925	0.914, 0.935	0.932	0.774	0.880	0.482
F5 Conflicts with Family Wishes	11	0.963	0.959, 0.968	0.960	0.689	0.830	0.427

*Note: k* = number of items; *α* = Cronbach’s alpha; Max *r* = the highest absolute correlation between the dimension and any other dimension. Values for *α* and its confidence interval were computed in the pooled sample (*N* = 540); the interval is a percentile interval from 10,000 bootstrap resamples. CR, AVE, √AVE, and Max *r* derive from the five‐factor intensity measurement model fitted in the confirmatory subsample (*n* = 242), which showed excellent fit: *χ* ^2^(655) = 731.00, *p* = 0.021; *χ* ^2^/df = 1.12; CFI = 0.991; TLI = 0.990; RMSEA = 0.022, 90% CI [0.009, 0.030]; and SRMR = 0.034. All 38 items were retained; items MDS‐37 and MDS‐38 were examined individually and retained on psychometric grounds, as described in Section 3.4. The Fornell–Larcker criterion was satisfied for every dimension, because √AVE exceeded Max *r* in each row.

Abbreviations: √AVE, square root of the average variance extracted; AVE, average variance extracted; CI, confidence interval; CR, composite reliability.

### 3.5. Measurement Invariance

Configural invariance across hospital type was examined by estimating the five‐factor model separately within each group. Fit was excellent in both: educational hospitals (*n* = 275) yielded CFI = 0.996, TLI = 0.995, RMSEA = 0.015 [0.000, 0.025], and SRMR = 0.031; noneducational hospitals (*n* = 265) yielded CFI = 0.993, TLI = 0.993, RMSEA = 0.019 [0.005, 0.027], and SRMR = 0.033. All 38 loadings were significant in both groups (*p* < 0.001), every item loaded on its intended factor, and the pattern of loadings was similar across groups (mean absolute difference = 0.09; largest difference 0.20 for MDS‐07). Interfactor correlations were comparably moderate in both groups (educational 0.274–0.504; noneducational 0.277–0.535; and largest between‐group difference 0.116). Configural invariance is therefore supported: the same five‐factor configuration holds in both hospital types (Supporting Table S4, Panel C).

Metric and scalar invariance were not formally tested. Establishing these levels requires constrained multigroup estimation, and with 38 indicators and 5 factors, the number of parameters relative to group size (*n* = 275 and 265) is insufficient for stable comparison of nested models. Consequently, although the configuration of the model is common to both groups, the equivalence of loadings and intercepts has not been demonstrated, and mean scores should not be compared between hospital types on the basis of the present evidence. The same limitation applies to gender and ED experience strata, for which no invariance testing was undertaken.

### 3.6. Concurrent Criterion‐Related Validity

Hierarchical regression indicated that the five dimensions accounted for substantial additional variance beyond demographic and occupational covariates: Δ*R* ^2^ = 0.494 for emotional exhaustion, *F*(5, 526) = 108.77, *p* < 0.001; Δ*R* ^2^ = 0.405 for depersonalization, *F*(5, 526) = 72.19, *p* < 0.001; and Δ*R* ^2^ = 0.466 for turnover intention, *F*(5, 526) = 93.70, *p* < 0.001. Total variance explained by the full models was 52.2%, 41.0%, and 47.7%, respectively. Notably, the demographic and occupational covariates entered at step one accounted for almost no variance in any outcome (*R* ^2^ = 0.029, 0.005, and 0.011), and were nonsignificant for depersonalization (*p* = 0.941) and turnover intention (*p* = 0.649). Communication and Autonomy Conflicts showed the strongest unique association with every outcome (*β* = 0.274–0.341, all *p* < 0.001), followed consistently by Systemic and Economic Constraints (*β* = 0.212–0.221, all *p* < 0.001). Conflicts with Family Wishes showed the weakest associations and did not reach significance for depersonalization (*β* = 0.054, *p* = 0.147). Because all measures were collected concurrently, these coefficients index association and do not establish direction of influence. Dimension‐level results are summarized in Table 4, with full model statistics in Supporting Table S6.

**TABLE 4:** Item length, internal consistency, and descriptive statistics for the five moral distress dimensions.

Dimension	*k*	*α*	Intensity *M*	Intensity SD	Frequency *M*	Frequency SD
F1 Communication and Autonomy Conflicts	10	0.958	3.49	1.52	2.56	1.25
F2 Unprofessional and Futile Care	8	0.955	3.42	1.57	2.42	1.30
F3 Systemic and Economic Constraints	5	0.941	3.28	1.73	2.31	1.40
F4 Working with Incompetent Colleagues	4	0.925	3.58	1.66	2.53	1.36
F5 Conflicts with Family Wishes	11	0.963	3.15	1.57	2.16	1.20

*Note: N* = 540. *k* = number of items contributing to the dimension within the 38‐item instrument; *α* = Cronbach’s alpha; *M* = mean. Intensity and frequency are reported as item means on 0–6 response scales so that dimensions of differing length are directly comparable. Dimensions are listed in the order in which they emerged from the exploratory analysis. Associations of these dimensions with burnout and turnover intention are reported in Table 5.

Abbreviation: SD, standard deviation.

Friedman testing indicated statistically significant differences among dimension intensity scores, *χ* ^2^(4) = 33.35, *p* < 0.001, but the effect size was negligible (Kendall’s *W* = 0.015), indicating that the five dimensions operated at broadly comparable moderate intensity. Item means spanned only 0.43 scale points, from 3.15 for Conflicts with Family Wishes to 3.58 for Working with Incompetent Colleagues. A unidimensional model, in which all 38 items were specified as indicators of a single latent factor, fitted very poorly: *χ* ^2^(665) = 5652.00, *p* < 0.001; *χ* ^2^/df = 8.50; CFI = 0.415; TLI = 0.381; RMSEA = 0.176 [0.172, 0.180]; and SRMR = 0.172. The deterioration relative to the five‐factor solution was substantial (ΔCFI = 0.576; ΔRMSEA = 0.154) and the chi‐square difference was significant, Δ*χ* ^2^(10) = 4921.00, *p* < 0.001. These results provide strong evidence that moral distress in this sample cannot be adequately represented as a single underlying dimension.

**TABLE 5:** Hierarchical regression of burnout and turnover intention on the five moral distress intensity dimensions.

Outcome and predictor	*β*	95% CI	*p*
Emotional exhaustion			
F1 Communication and Autonomy Conflicts	0.341	0.271, 0.410	< 0.001
F2 Unprofessional and Futile Care	0.185	0.111, 0.259	< 0.001
F3 Systemic and Economic Constraints	0.221	0.149, 0.293	< 0.001
F4 Working with Incompetent Colleagues	0.142	0.070, 0.214	< 0.001
F5 Conflicts with Family Wishes	0.081	0.015, 0.147	0.02
Step 1 *R* ^2^ (covariates only)	0.029		
Δ*R* ^2^ for Step 2	0.494		
Total *R* ^2^	0.522		
Δ*F* (5, 526) for Step 2	108.77		< 0.001
Depersonalization			
F1 Communication and Autonomy Conflicts	0.313	0.236, 0.390	< 0.001
F2 Unprofessional and Futile Care	0.177	0.094, 0.259	< 0.001
F3 Systemic and Economic Constraints	0.212	0.132, 0.292	< 0.001
F4 Working with Incompetent Colleagues	0.113	0.034, 0.193	0.005
F5 Conflicts with Family Wishes	0.054	−0.019, 0.128	0.15
Step 1 *R* ^2^ (covariates only)	0.005		
Δ*R* ^2^ for Step 2	0.405		
Total *R* ^2^	0.410		
Δ*F* (5, 526) for Step 2	72.19		< 0.001
Turnover intention			
F1 Communication and Autonomy Conflicts	0.274	0.201, 0.346	< 0.001
F2 Unprofessional and Futile Care	0.211	0.133, 0.289	< 0.001
F3 Systemic and Economic Constraints	0.216	0.141, 0.292	< 0.001
F4 Working with Incompetent Colleagues	0.161	0.085, 0.236	< 0.001
F5 Conflicts with Family Wishes	0.091	0.022, 0.160	0.010
Step 1 *R* ^2^ (covariates only)	0.011		
Δ*R* ^2^ for Step 2	0.466		
Total *R* ^2^	0.477		
Δ*F* (5, 526) for Step 2	93.70		< 0.001

*Note: N* = 540. *β* = standardized regression coefficient from step two of a hierarchical multiple regression. Step one contained age, gender, marital status, education, hospital type, emergency department experience, employment status, and shift type; step two added the five moral distress intensity dimensions simultaneously. Exact *p* values are reported; *p* values below .001 are shown as < 0.001. Coefficients index concurrent association in cross‐sectional data and do not establish direction of influence. Increments were computed from unrounded estimates, so a rounded step‐one *R* ^2^ and Δ*R* ^2^ may not sum exactly to the rounded total *R* ^2^. Clinical interpretations of each dimension, exemplar item content, and candidate intervention targets are provided in Supporting Tables S7 and S8.

Abbreviation: CI, confidence interval.

## 4. Discussion

This study examined the dimensional structure of moral distress among emergency nurses in a non‐Western context using a two‐stage split‐half design with concurrent criterion‐related validation. A five‐factor structure emerged consistently across both intensity and frequency subscales, was cross‐validated in an independent subsample with excellent fit, and showed substantial associations with burnout and turnover intention. A unidimensional alternative fitted very poorly.

### 4.1. The Case for Dimensionality

The principal finding is that a five‐factor representation fitted these data substantially better than a unidimensional alternative. The very poor fit of the single‐factor model (CFI = 0.415; TLI = 0.381; and RMSEA = 0.176) indicates that the ethical conflicts sampled by this instrument do not reduce to a single underlying quantity in this population. The dimensions identified—constrained communication and autonomy, unprofessional and futile care, systemic and economic constraint, incompetent colleagues, and conflict with family wishes—correspond to recognizably distinct classes of constraint in Jameton’s sense.

The practical implication concerns interpretation rather than mere model fit. Two nurses may record identical aggregate scores while differing entirely in the source of their distress: one constrained by resource unavailability and the other by hierarchical silencing. An aggregate score renders these cases indistinguishable, and any organizational response derived from it is correspondingly undirected. This is not a novel theoretical claim—the heterogeneity of moral distress has long been recognized conceptually—but it is infrequently tested empirically against a unidimensional alternative, and our results suggest that the assumption embedded in aggregate scoring does not hold here.

### 4.2. Constrained Professional Voice as the Strongest Correlate

Communication and Autonomy Conflicts showed the strongest unique association with emotional exhaustion (*β* = 0.341), depersonalization (*β* = 0.313), and turnover intention (*β* = 0.274), in each case exceeding every other dimension. This dimension captures the experience of recognizing an appropriate course of action while lacking the standing to act on it—being unable to voice concern, having assessments disregarded, carrying out orders judged unsafe. That it should be the dimension most strongly associated with adverse outcomes is consistent with the constraint‐centered formulation since these items describe constraint in its most direct form.

This finding converges with the qualitative literature. Wolf et al. [2], in interviews with emergency nurses in the United States, found that participants described moral distress as environment‐driven rather than incident‐driven, with constrained agency a recurrent theme—a striking convergence given the substantial differences between the two healthcare systems. It also corresponds partially with Akbarzadeh et al. [15], whose Persian adaptation of the Moral Distress Scale–Revised in pediatric nurses yielded a “lack of moral courage” factor that overlaps conceptually with our F1, though their formulation locates the deficit in the individual whereas ours locates it in the interactional structure—a difference in emphasis that may itself warrant investigation.

A divergence is also instructive. In Western intensive care samples, futile care typically emerges as the dominant source of distress, whereas here, it ranked below constrained voice. We attribute this tentatively to setting rather than to culture: emergency nurses rarely manage prolonged end‐of‐life trajectories and their exposure to sustained futile treatment is correspondingly lower than that of ICU colleagues. This interpretation is testable and would be resolved by comparing ED and ICU nurses within the same system.

The direction of these associations cannot be determined from these data. Emotionally exhausted nurses may perceive and report communication conflicts more readily, constrained voice may contribute to exhaustion, and reciprocal processes are plausible. The moral residue model would predict accumulation over time, which is consistent with our findings but not established by them.

### 4.3. Concurrent Criterion‐Related Validity

Beyond demographic and occupational covariates, the five dimensions accounted for an additional 49.4% of variance in emotional exhaustion, 40.5% in depersonalization, and 46.6% in turnover intention. The contrast with the covariate block is striking: age, gender, marital status, education, ED experience, employment status, shift type, and hospital type together accounted for less than 3% of variance in any outcome and were nonsignificant for two of the three. This pattern suggests that what nurses experience in their work is far more closely associated with burnout and turnover intention than who they are demographically. These values nonetheless index concurrent association rather than prediction: because all measures were obtained at a single time point, the analysis establishes concurrent criterion‐related validity and cannot adjudicate the direction of influence. It is equally plausible that emotionally exhausted nurses perceive and report communication conflicts more readily as it is that such conflicts contribute to exhaustion, and reciprocal processes are likely. Longitudinal designs are required to establish temporal ordering.

These magnitudes may be compared with the surrounding literature. Alshammari et al. [8] accounted for 67.9% of burnout variance among Saudi emergency nurses using sociodemographic and occupational predictors, and Wu et al. [10] identified hospital ethical climate and moral resilience as mediators of the moral distress–burnout relationship among Chinese emergency nurses. Our contribution is complementary rather than competing: those studies model person‐level and climate‐level predictors, whereas we show that disaggregating the moral distress construct itself yields explanatory value beyond an aggregate score. Taken together, the three sets of findings suggest that individual characteristics, organizational climate, and the internal structure of distress each contribute distinct information.

### 4.4. Measurement Invariance and Its Limits

Measurement invariance was examined only at the configural level. The five‐factor model fitted excellently when estimated separately in educational and noneducational hospitals, with loadings of similar magnitude and comparable interfactor correlations, indicating that the same configuration underlies responses in both settings. This is a meaningful finding in its own right: the dimensional architecture is not an artefact of one organizational context.

Configural invariance is nonetheless the weakest of the three conventional levels, and the practical implication should be stated plainly: managers should not use dimension mean scores to benchmark units, hospital types, or staff groups against one another on the basis of the present evidence. Such comparisons presuppose scalar invariance, which was not tested and which the available subgroup sizes could not support for a model of this complexity. The instrument is presently suited to examining relationships among constructs within a population, and to monitoring change within a group over time, rather than to comparing levels between groups. Establishing metric and scalar invariance in a larger sample is a prerequisite for that application and a clear priority for subsequent work.

### 4.5. Interpreting the Family Conflict Dimension

Conflicts with Family Wishes emerged as a distinct dimension but recorded the lowest mean intensity. In Western bioethics, family override of patient preference is typically a prominent source of distress, and the contrast invites explanation. We previously advanced a cultural account; on reflection, our data support no firm conclusion and at least three explanations are consistent with the finding.

The first is cultural congruence: family‐centered decision‐making is normative in Iranian practice and aligned with Islamic bioethical reasoning [14], so family involvement may not be construed as a violation and may generate correspondingly less distress. The second is differential exposure: family conflicts may simply arise less frequently in the ED than in settings with prolonged patient relationships, in which case lower intensity reflects the distribution of encounters rather than any cultural buffering. The third is habituation: nurses practising within a system where family override is routine may have normalized the experience, in which case the appropriate interpretation is suppressed rather than absent distress—a possibility with quite different practical implications since normalized distress may still contribute to moral residue while remaining invisible to self‐report.

The present design cannot distinguish these accounts. Doing so would require direct measurement of cultural values and religiosity, comparison of ED with longer‐stay settings within the same system, and cross‐national comparison using an invariant instrument. Until such evidence exists, the cultural interpretation should be treated as a hypothesis rather than a finding.

### 4.6. Comparable Intensity and Distinct Structure

Although the Friedman test indicated statistically significant differences among dimension intensities, the effect size was negligible (Kendall’s *W* = 0.015), and item means spanned only 0.43 scale points. This should not be mistaken for evidence of unidimensionality. Equivalent average intensity is compatible with entirely distinct structure and distinct outcome associations, both of which we observed. A particularly instructive dissociation emerges from comparing intensity rankings with outcome associations: Working with Incompetent Colleagues recorded the highest mean intensity (3.58) yet showed among the weakest unique associations with outcomes (*β* = 0.113 to 0.161), whereas Communication and Autonomy Conflicts ranked second in intensity (3.49) but showed the strongest associations throughout (*β* = 0.274 to 0.341). How distressing a class of ethical conflict feels is, therefore, a poor guide to how strongly it relates to burnout and turnover intention. This dissociation is invisible to an aggregate score and constitutes a practical argument for dimensional measurement: organizations attending only to the most intensely felt conflicts might overlook those most closely associated with workforce attrition.

### 4.7. Positioning Within Adjacent Literature

#### 4.7.1. Moral Injury

Our findings sit within a broader literature positioning moral distress on a continuum toward moral injury. Rimon and Shelef [11], reviewing medical personnel and first responders, report prevalence estimates from 4.1% to 69.4% and identify organizational constraint, traumatic exposure, and absence of support as recurrent antecedents. Notably, the dimension most strongly associated with adverse outcomes in our data corresponds to the betrayal‐based pathway described in that literature—injury arising from the conduct of trusted others or institutions—rather than the perpetration‐based pathway. If this correspondence holds, the chronic constraint of professional voice may warrant attention as a candidate antecedent of moral injury and not solely of burnout.

#### 4.7.2. Ethical Climate

Wu et al. [10] found hospital ethical climate to mediate the relationship between moral distress and burnout among Chinese emergency nurses. Our F1 and F3 dimensions overlap substantially with constructs measured by ethical climate instruments, particularly regarding whether concerns can be raised and how resource decisions are made. This raises a measurement question we cannot resolve here: whether these dimensions index individually experienced distress or perceptions of a shared organizational property. Their small but nonzero hospital‐level ICCs (0.034) suggest predominantly individual variance, but designs capable of separating the two levels would be informative.

#### 4.7.3. Moral Resilience

Our exclusion of nurses who had recently undertaken ethics or moral resilience training protected internal validity but restricts generalization: the estimates apply to an untrained population and may overstate distress relative to settings where such programs are routine. Given evidence that moral resilience moderates the distress–burnout relationship, its omission as a measured variable is a limitation, and incorporating it would clarify whether the primacy of F1 persists among nurses with greater resilience resources.

#### 4.7.4. Organizational Ethics

The inclusion of error‐reporting pressure within the systemic constraints dimension connects these findings to the just‐culture literature. That reporting pressure loaded with resource shortage and cost‐driven limitation, rather than forming a separate factor, is consistent with treatments of reporting culture as an organizational property arising from the same institutional conditions that produce resource constraint, rather than as an individual behavioral disposition.

### 4.8. Contribution to Measurement and Conceptual Refinement

The contribution of this study is best characterized as psychometric refinement and contextual extension rather than theoretical innovation. It does not propose a new model of moral distress; the construct remains as formulated by Jameton [3] and elaborated by Epstein and Hamric [6] and Morley et al. [5]. What the study adds is threefold: an explicit empirical test of dimensionality against a unidimensional alternative, which is seldom reported; evidence that dimension‐level scores account for variance in burnout and turnover intention beyond an aggregate score; and extension of dimensional measurement into a non‐Western emergency nursing context in which the construct has been underexamined. Multidimensional treatments of moral distress are not themselves new, and we do not claim priority for the idea.

### 4.9. Limitations

Several limitations qualify these findings. First, and most consequentially, the cross‐sectional design precludes causal inference. The associations between moral distress dimensions, burnout, and turnover intention are concurrent, and three interpretations remain open: that constrained practice contributes to burnout, that burnout alters the perception and reporting of constraint, and that both are sustained by common organizational conditions. Nothing in these data adjudicates between them, and the incremental variance statistics should not be read as evidence of predictive validity in the strict sense.

Second, although all dimensions met conventional psychometric criteria, *F*1 and *F*5 are the broadest in content coverage, spanning ten and eleven items, respectively. Breadth of coverage is necessarily traded against homogeneity, and a more narrowly specified alternative might yield higher average variance extracted at the cost of conceptual comprehensiveness. We regard this as a design tension rather than a defect, but note it for transparency. We also note that MDS‐37 and MDS‐38, while psychometrically adequate, were the items most frequently queried during cognitive interviewing and may merit rewording in future revisions of the scale.

Third, generalization beyond the study context should be cautious. The sample was drawn from six public hospitals in three cities of western Iran within a single national healthcare system. Features of that system—physician–nurse authority gradients, family‐centered decision norms, and macroeconomic constraint on supply availability—may not transfer to other settings, and the structure requires cross‐national replication before it can be treated as generalizable. We have removed claims of global applicability made in the original submission.

Fourth, measurement invariance was established only at the configural level and only across the hospital type. Metric and scalar invariance were not tested, and no invariance analysis was undertaken across gender or ED experience; the instrument, therefore, cannot presently support comparison of scores between groups. Fifth, all measures were self‐reported and collected concurrently; although the design included no procedural remedies for common method variance, its influence cannot be excluded. Sixth, although intraclass correlations were small, the limited number of clusters precluded multilevel modeling, so standard errors may not fully account for institutional clustering. Seventh, the cultural interpretation of the family conflict dimension remains speculative because cultural values and religiosity were not directly measured. Eighth, the exclusion of nurses with recent ethics training restricts the findings to an untrained population. Finally, objective outcomes—actual turnover, medication errors, patient safety indicators, and human resources records—were not available, and turnover intention is an imperfect proxy for turnover behavior.

### 4.10. Implications for Measurement and Practice

For measurement, three implications follow. Dimension‐level scores should be reported alongside or instead of aggregate totals since the aggregate discards information that is associated with outcomes. Group mean comparisons should not be undertaken until measurement invariance has been established in an adequately powered sample. And the absence of a validated cutoff should be respected: no threshold on this instrument currently distinguishes clinically significant from ordinary distress, and deriving one would require calibration against an external criterion such as diagnostic interview or prospective resignation, using ROC analysis in a longitudinal design. Until then, the instrument is suited to comparing relationships and monitoring change within groups, not to individual screening.

For practice, the dimensional structure suggests that organizational responses might be matched to the specific conflicts prevalent in a given unit, rather than delivered as undifferentiated wellbeing provision. Supporting Table S8 sets out candidate targets mapped to each dimension. We emphasize that these are hypotheses generated by the dimensional structure and the pattern of associations; no intervention was tested in this study, and each requires prospective evaluation. Given that constrained professional voice showed the strongest associations across all three outcomes, mechanisms that restore nursing input to clinical decisions may merit priority consideration, but this inference rests on cross‐sectional association and should be treated accordingly.

## 5. Conclusions

Among emergency nurses in six Iranian public hospitals, moral distress was better represented as a multidimensional than a unidimensional construct. A five‐factor structure emerged consistently across intensity and frequency ratings, was cross‐validated in an independent subsample, and accounted for substantial variance in burnout and turnover intention beyond demographic and occupational characteristics. Constrained professional voice was the dimension most strongly associated with all three outcomes, identifying it as a candidate priority for organizational attention.

These findings qualify the routine use of aggregate moral distress scores and offer a dimensional framework for more differentiated monitoring. Three caveats bound their application. The associations are concurrent and do not establish direction of influence. Measurement invariance has not been established, so the instrument cannot presently support comparison of scores between groups. And the evidence derives from a single national healthcare system, so cross‐national replication is required before the structure can be considered generalizable. Longitudinal designs incorporating objective turnover, adequately powered invariance testing, and studies measuring cultural values directly represent the most informative next steps.

## Author Contributions

Fatemeh Alidadi: conceptualization, methodology, investigation, data curation, formal analysis, writing–original draft, and project administration. Vahid Zamanzadeh: conceptualization, methodology, supervision, writing–review and editing, and correspondence. Fatemeh Monjazebi: methodology, validation, and writing–review and editing. Fataneh Ghadirian: investigation, resources, and writing–review and editing.

## Funding

No external funding was received.

## Disclosure

Written permission to use the 38‐item Moral Distress Scale was granted by Professor Mary C. Corley, the lead developer of the instrument, on 30 July 2026. Her permission email is provided as Supporting File A. Professor R. K. Elswick Jr. (Virginia Commonwealth University), second author of Corley et al. [21], and Professor Beth Epstein (University of Virginia) were also consulted and raised no objection.

## Ethics Statement

This study was approved by the Research Ethics Committee of Shahid Beheshti University of Medical Sciences (IR.SBMU.PHARMACY.REC.1403.052).

## Consent

Written informed consent was obtained from all participants.

## Conflicts of Interest

The authors declare no conflicts of interest.

## Endnotes

^1^Principal Axis Factoring estimates only the variance shared among items (common variance); percentages of explained variance are therefore expressed as a proportion of extracted common variance. These figures are not directly comparable with the total‐variance percentages produced by Principal Components Analysis, which are typically larger because PCA analyses common and unique variance together.

## Supporting Information

Additional supporting information can be found online in the Supporting Information section.

## Supporting information


**Supporting Information** Additional supporting information may be found in the online version of this article at the publisher’s website. The following supporting information is provided with this article: Supporting File A. Written permission from Professor Mary C. Corley, lead developer of the Moral Distress Scale, authorizing use of the 38‐item instrument in this study (email dated 30 July 2026). Supporting Table S1. Item analysis for the Moral Distress Scale intensity subscale (*N* = 540), reporting for each of the 38 items the mean, standard deviation, difficulty index, skewness, kurtosis, corrected item–total correlation, and Cronbach’s alpha if the item were deleted. Supporting Table S2. Complete rotated pattern matrix for the intensity subscale in the exploratory subsample (*n* = 298), reporting all primary loadings, all cross‐loadings, and item communalities. Supporting Table S3. Factor correlation matrices, eigenvalues with parallel‐analysis comparison values, and Tucker’s congruence coefficients between the intensity and frequency solutions. Supporting Table S4. Confirmatory factor analysis in the confirmatory subsample (*n* = 242): standardized loadings for all 38 items, comparison of the five‐factor and unidimensional models, and configural invariance across hospital type. Supporting Table S5. Reliability and convergent validity by dimension, reporting the number of items, Cronbach’s alpha, composite reliability, and average variance extracted for each of the five dimensions and for the total scale. Supporting Table S6. Full hierarchical regression models predicting emotional exhaustion, depersonalization, and turnover intention (*N* = 540), including all step‐one covariates and complete model statistics. Supporting Table S7. Clinical interpretation and exemplar item content for the five moral distress dimensions. Supporting Table S8. Candidate intervention targets mapped to the moral distress dimensions, with the rationale and evidential status of each. 5_STROBE_Checklist_1438300.

## Data Availability

Deidentified data are available from the corresponding author upon reasonable request.
