# Supplementary material for: Dimensionality of Moral Distress Among Emergency Nurses in a Non‐Western Context: A Two‐Stage Psychometric Validation
Source: J Nurs Manag. 2026 Oct 15;2026:1438300. doi: 10.1155/jonm/1438300 (PMC13670431; doi:10.1155/jonm/1438300)
Supplement: Supplementary file 1 [file JONM-2026-1438300-s001.zip › 5_STROBE_Checklist_1438300.docx]

**STROBE Statement — Checklist for Cross-Sectional Studies**

*Dimensionality of Moral Distress among Emergency Nurses in a Non-Western Context: A Two-Stage Psychometric Validation*

*Journal of Nursing Management — Manuscript ID 1438300 — Revision 2*

| **Title and abstract** | | |
| --- | --- | --- |
| **1 (a)** | Indicate the study’s design with a commonly used term in the title or the abstract | Title; Abstract (Design): “two-stage split-half psychometric study”; cross-sectional data collection. |
| **1 (b)** | Provide in the abstract an informative and balanced summary of what was done and what was found | Abstract: Background, Objectives, Design, Methods, Results, Conclusions, and Implications for Nursing Management. |
| **Introduction** | | |
| **2** | Explain the scientific background and rationale for the investigation being reported | Section 1, paragraphs 1–6. |
| **3** | State specific objectives, including any prespecified hypotheses | Section 1, final paragraph (objectives 1–5). |
| **Methods** | | |
| **4** | Present key elements of study design early in the paper | Section 2.1. |
| **5** | Describe the setting, locations, and relevant dates, including periods of recruitment, exposure, follow-up, and data collection | Section 2.1: 1 June to 30 September 2025; six university-affiliated public hospitals in three cities in western Iran. |
| **6 (a)** | Give the eligibility criteria, and the sources and methods of selection of participants | Section 2.2: inclusion and exclusion criteria; total-population (census) sampling at participant level; purposive selection of the six hospitals. |
| **6 (b)** | Matched studies — give matching criteria and number of exposed and unexposed | Not applicable (not a matched design). |
| **7** | Clearly define all outcomes, exposures, predictors, potential confounders, and effect modifiers; give diagnostic criteria, if applicable | Section 2.4: Moral Distress Scale, Maslach Burnout Inventory–Human Services Survey, and turnover intention measure. Section 2.6: covariates entered at step one of the hierarchical regression. |
| **8** | For each variable of interest, give sources of data and details of methods of assessment (measurement); describe comparability of assessment methods if there is more than one group | Section 2.4 (all three instruments, with scaling and reliability); Section 2.5 (six-stage translation and cultural adaptation). |
| **9** | Describe any efforts to address potential sources of bias | Section 2.2: non-response bias assessed by chi-square, t tests, and TOST equivalence testing. Section 2.5: cultural and translation bias addressed through expert committee review and cognitive interviewing. Section 4.9: common method variance acknowledged as a limitation. |
| **10** | Explain how the study size was arrived at | Section 2.2: census sampling; respondent-to-item ratio 7.8:1 for the exploratory stage and n = 242 for the confirmatory stage, against a conventional minimum of 200 (Schreiber et al., 2006). |
| **11** | Explain how quantitative variables were handled in the analyses; if applicable, describe which groupings were chosen and why | Section 2.6: continuous variables retained as continuous; item analysis, Principal Axis Factoring with Promax rotation, confirmatory factor analysis, and hierarchical regression described in sequence with the decision rules applied at each stage. |
| **12 (a)** | Describe all statistical methods, including those used to control for confounding | Section 2.6: Principal Axis Factoring; parallel analysis with 1,000 permutations; maximum likelihood confirmatory estimation; hierarchical regression with demographic and occupational covariates entered at step one. |
| **12 (b)** | Describe any methods used to examine subgroups and interactions | Section 2.6 and Section 3.5: configural invariance examined across hospital type by estimating the model separately within each group. Metric and scalar invariance were not tested; the rationale is given in Sections 3.5 and 4.4. |
| **12 (c)** | Explain how missing data were addressed | Section 2.2: listwise deletion, with the number and reasons for deletions reported separately for each subsample; deleted and retained cases compared on all measured characteristics. |
| **12 (d)** | If applicable, describe analytical methods taking account of sampling strategy | Section 2.6: hospital-level and city-level intraclass correlations inspected; multilevel modelling not feasible given the small number of clusters, so hospital type was entered as a covariate. The clustering limitation is stated in Section 4.9. |
| **12 (e)** | Describe any sensitivity analyses | Section 3.6: a unidimensional model was estimated and compared against the five-factor solution (Supplementary Table S4, Panel B). Section 3.5: the model was re-estimated separately within educational and non-educational hospitals (Supplementary Table S4, Panel C). |
| **Results** | | |
| **13 (a)** | Report numbers of individuals at each stage of the study | Section 2.2, Section 3.1, and Figure 1: 620 eligible; 568 consented; randomised to 312 and 256; 298 and 242 after listwise deletion; pooled analytic sample 540. |
| **13 (b)** | Give reasons for non-participation at each stage | Section 2.2: 52 non-respondents, with demographic data available for 48; reasons for all 28 deletions reported separately by subsample. |
| **13 (c)** | Consider use of a flow diagram | Figure 1. |
| **14 (a)** | Give characteristics of study participants and information on exposures and potential confounders | Section 3.1 and Table 2. |
| **14 (b)** | Indicate the number of participants with missing data for each variable of interest | Section 2.2: 151 missing cells across the 28 deleted cases (2–8 per case); no missing values on any Moral Distress Scale item. |
| **14 (c)** | Cohort study — summarise follow-up time | Not applicable (cross-sectional design). |
| **15** | Report numbers of outcome events or summary measures | Section 3.2: means, standard deviations, and proportions above the scale midpoint, with the midpoint labelled as an arbitrary descriptive reference. Sections 3.3–3.6 and Tables 3 and 4. |
| **16 (a)** | Give unadjusted estimates and, if applicable, confounder-adjusted estimates and their precision; make clear which confounders were adjusted for and why they were included | Section 3.6, Table 4, and Supplementary Table S6: standardised coefficients from step two of the hierarchical regression, adjusted for age, gender, marital status, education, ED experience, employment status, shift type, and hospital type. |
| **16 (b)** | Report category boundaries when continuous variables were categorized | Not applicable: continuous variables were retained as continuous in all models. Demographic categories used descriptively in Table 2 are defined there. |
| **16 (c)** | If relevant, consider translating estimates of relative risk into absolute risk for a meaningful time period | Not applicable (no risk estimates reported). |
| **17** | Report other analyses done — eg analyses of subgroups and interactions, and sensitivity analyses | Section 3.5: configural invariance across hospital type. Section 3.6: Friedman test with Kendall’s W, and comparison against a unidimensional model. |
| **Discussion** | | |
| **18** | Summarise key results with reference to study objectives | Sections 4.1–4.3. |
| **19** | Discuss limitations of the study, taking into account sources of potential bias or imprecision; discuss both direction and magnitude of any potential bias | Section 4.9: nine limitations, beginning with the cross-sectional design and the reverse and reciprocal pathways it leaves open; also breadth of F1 and F5, generalisability, invariance, common method variance, clustering, unmeasured cultural values, exclusion of ethics-trained nurses, and the absence of objective turnover data. |
| **20** | Give a cautious overall interpretation of results considering objectives, limitations, multiplicity of analyses, results from similar studies, and other relevant evidence | Sections 4.2–4.8: associational language throughout; three competing explanations advanced for the F5 finding in Section 4.5; contribution framed as psychometric refinement rather than theoretical innovation in Section 4.8. |
| **21** | Discuss the generalisability (external validity) of the study results | Sections 4.9 and 5: findings limited to Iranian public-sector emergency nursing; cross-national replication required. |
| **Other information** | | |
| **22** | Give the source of funding and the role of the funders for the present study and, if applicable, for the original study on which the present article is based | Declarations (Funding): no external funding was received. Declarations (Competing interests): none declared. |

**Note.** *Section, table, and figure references are to the clean revised manuscript (Revision 2).*
