# Supplementary material for: Dimensionality of Moral Distress Among Emergency Nurses in a Non‐Western Context: A Two‐Stage Psychometric Validation
Source: J Nurs Manag. 2026 Oct 15;2026:1438300. doi: 10.1155/jonm/1438300 (PMC13670431; doi:10.1155/jonm/1438300)
Supplement: Supplementary file 1 [file JONM-2026-1438300-s001.zip › 0FSX.pdf]

Supplementary Material

Dimensionality of Moral Distress among Emergency Nurses in a Non-Western Context: A Two-Stage Psychometric Validation

Journal of Nursing Management — Manuscript ID 1438300

Supplementary Table S1. Item analysis for the Moral Distress Scale intensity subscale (N = 540)

Item	Abbreviated content	Factor	M	SD	Difficulty	Scale M if deleted	Scale var. if deleted	Corrected item–total r	α if deleted
MDS-01	Unable to voice ethical concern because of hierarchy	F1	3.57	1.86	0.59	124.09	1727.4	0.615	0.954
MDS-02	Physician ignores nursing assessment	F1	3.53	1.85	0.59	124.14	1727.7	0.606	0.954
MDS-03	Carrying out orders against clinical judgement	F1	3.64	1.91	0.61	124.08	1726.7	0.607	0.954
MDS-04	Professional judgement not valued in care decisions	F1	3.58	1.79	0.60	124.20	1726.8	0.618	0.954
MDS-05	Unable to question inappropriate treatment decisions	F1	3.50	1.81	0.58	124.22	1731.0	0.593	0.955
MDS-06	Being excluded from ethically important discussions	F1	3.47	1.84	0.58	124.24	1730.1	0.602	0.954
MDS-07	Pressure to follow decisions perceived as unsafe	F1	3.33	1.79	0.56	124.26	1737.0	0.555	0.955
MDS-08	Difficulty advocating for patient preferences	F1	3.45	1.84	0.58	124.27	1729.5	0.602	0.954
MDS-09	Limited opportunity to discuss prognosis or goals	F1	3.40	1.82	0.57	124.26	1734.6	0.572	0.955
MDS-10	Lack of respect for nursing professional role	F1	3.23	1.73	0.54	124.45	1741.6	0.539	0.955
MDS-11	Continuing treatment perceived as medically futile	F2	3.51	1.81	0.58	124.24	1726.3	0.626	0.954
MDS-12	Providing aggressive care with little expected benefit	F2	3.62	1.83	0.60	124.29	1723.8	0.639	0.954
MDS-13	Performing non-beneficial procedures to satisfy others	F2	3.56	1.78	0.59	124.27	1728.4	0.609	0.954
MDS-14	Continuing life-sustaining treatment despite poor prognosis	F2	3.51	1.85	0.59	124.28	1730.3	0.588	0.955
MDS-15	Participating in care viewed as prolonging suffering	F2	3.44	1.87	0.57	124.30	1725.0	0.625	0.954
MDS-16	Assisting with care judged professionally inappropriate	F2	3.56	1.90	0.59	124.25	1725.6	0.609	0.954
MDS-17	Witnessing unprofessional care practices	F2	3.46	1.78	0.58	124.36	1731.9	0.593	0.955
MDS-18	Continuing questionable intervention despite ethical concern	F2	3.46	1.79	0.58	124.36	1727.0	0.636	0.954
MDS-19	Providing suboptimal care because of resource shortage	F3	3.31	1.88	0.55	124.36	1729.0	0.564	0.955
MDS-20	Patient care limited by economic pressures	F3	3.21	1.97	0.54	124.43	1723.2	0.575	0.955
MDS-21	Inability to provide needed care because of cost	F3	3.31	1.86	0.55	124.42	1731.4	0.552	0.955
MDS-22	Pressure to conceal or avoid reporting errors	F3	3.18	1.83	0.53	124.45	1729.6	0.570	0.955
MDS-23	Institutional constraints limiting ethically appropriate care	F3	3.19	1.87	0.53	124.51	1729.1	0.569	0.955
MDS-24	Working with incompetent healthcare providers	F4	3.70	1.93	0.62	124.15	1733.0	0.557	0.955
MDS-25	Colleagues fail to meet patient care needs	F4	3.60	1.86	0.60	124.17	1737.7	0.539	0.955
MDS-26	Working with clinically unsafe staff	F4	3.69	1.89	0.61	124.14	1731.5	0.568	0.955
MDS-27	Working with impaired or unreliable staff	F4	3.72	1.86	0.62	124.08	1735.3	0.555	0.955
MDS-28	Family excludes capable patient from decisions	F5	3.52	1.88	0.59	124.41	1725.2	0.591	0.955
MDS-29	Family requests aggressive care despite poor prognosis	F5	3.49	1.79	0.58	124.39	1728.5	0.592	0.955
MDS-30	Family wishes conflict with patient preferences	F5	3.44	1.89	0.57	124.37	1723.1	0.597	0.955
MDS-31	Family resists discussion of DNR or treatment limits	F5	3.50	1.81	0.58	124.45	1723.2	0.616	0.954
MDS-32	Religious or cultural objections complicate standard care	F5	3.34	1.88	0.56	124.51	1724.5	0.597	0.954
MDS-33	Family requests withholding information from patient	F5	3.47	1.85	0.58	124.46	1724.8	0.603	0.954
MDS-34	Family insists on treatment viewed as burdensome	F5	3.41	1.81	0.57	124.43	1728.2	0.586	0.955
MDS-35	Patient preference overridden by family decision	F5	3.37	1.80	0.56	124.51	1729.9	0.582	0.955

MDS-36	Difficulty balancing family wishes and patient welfare	F5	3.21	1.81	0.54	124.56	1725.9	0.621	0.954
MDS-37	Avoiding end-of-life discussion because of family conflict	F5	2.75	1.60	0.46	125.03	1744.5	0.549	0.955
MDS-38	Cultural preference conflict in end-of-life care	F5	2.74	1.62	0.46	125.07	1746.5	0.540	0.955

Note. N = 540. Difficulty index = item mean / maximum possible score (6). Corrected item–total correlations ranged from .539 to .639 (M = .588), all exceeding the .30 criterion and indeed the more stringent .50 threshold. Total-scale Cronbach's α = .956; no item deletion would have increased it. Items MDS-37 and MDS-38, identified during cognitive interviewing as potentially double-barrelled, showed the lowest item means (2.75) but acceptable item–total correlations (.549 and .540) and were retained.

Supplementary Table S2. Complete rotated pattern matrix, intensity subscale (EFA subsample, n = 298)

Item	Abbreviated content	F1	F2	F3	F4	F5	h^2
MDS-01	Unable to voice ethical concern because of hierarchy	.859 *	−.035	.016	.007	.009	.738
MDS-02	Physician ignores nursing assessment	.847 *	−.044	.076	−.056	.059	.739
MDS-03	Carrying out orders against clinical judgement	.863 *	.018	.016	−.036	.013	.749
MDS-04	Professional judgement not valued in care decisions	.786 *	.098	−.055	−.005	.053	.674
MDS-05	Unable to question inappropriate treatment decisions	.837 *	.037	−.002	−.025	.032	.723
MDS-06	Being excluded from ethically important discussions	.819 *	−.043	.036	−.026	.074	.691
MDS-07	Pressure to follow decisions perceived as unsafe	.815 *	−.024	−.030	.078	−.102	.647
MDS-08	Difficulty advocating for patient preferences	.847 *	−.019	−.013	.075	−.099	.712
MDS-09	Limited opportunity to discuss prognosis or goals	.801 *	.023	−.014	.018	−.020	.650
MDS-10	Lack of respect for nursing professional role	.804 *	.000	−.008	−.031	−.007	.618
MDS-11	Continuing treatment perceived as medically futile	.006	.851 *	.021	.008	−.001	.753
MDS-12	Providing aggressive care with little expected benefit	.051	.795 *	−.010	.016	.067	.712
MDS-13	Performing non-beneficial procedures to satisfy others	.027	.861 *	−.025	−.018	−.009	.721
MDS-14	Continuing life-sustaining treatment despite poor prognosis	−.111	.877 *	.043	−.050	.016	.714
MDS-15	Participating in care viewed as prolonging suffering	.043	.803 *	−.042	.036	.022	.681
MDS-16	Assisting with care judged professionally inappropriate	.012	.793 *	.064	.028	−.041	.692
MDS-17	Witnessing unprofessional care practices	−.050	.846 *	.071	−.045	.005	.716
MDS-18	Continuing questionable intervention despite ethical concern	.043	.885 *	−.083	.050	−.057	.758
MDS-19	Providing suboptimal care because of resource shortage	−.045	.055	.867 *	−.027	.020	.760
MDS-20	Patient care limited by economic pressures	.042	.007	.866 *	.017	−.028	.788
MDS-21	Inability to provide needed care because of cost	.019	−.027	.876 *	.025	−.013	.770
MDS-22	Pressure to conceal or avoid reporting errors	−.011	.004	.897 *	.012	−.009	.803
MDS-23	Institutional constraints limiting ethically appropriate care	.028	.010	.872 *	−.007	.022	.796
MDS-24	Working with incompetent healthcare providers	.031	.030	−.009	.834 *	−.009	.731
MDS-25	Colleagues fail to meet patient care needs	−.037	.018	.032	.867 *	−.012	.754
MDS-26	Working with clinically unsafe staff	.012	−.009	−.028	.860 *	.039	.747
MDS-27	Working with impaired or unreliable staff	.013	−.012	.028	.822 *	.023	.708
MDS-28	Family excludes capable patient from decisions	.009	−.073	−.015	.011	.899 *	.777
MDS-29	Family requests aggressive care despite poor prognosis	.088	.007	−.022	−.080	.852 *	.724
MDS-30	Family wishes conflict with patient preferences	−.039	−.057	.008	.043	.898 *	.789
MDS-31	Family resists discussion of DNR or treatment limits	−.005	.032	−.027	.052	.839 *	.741
MDS-32	Religious or cultural objections complicate standard care	−.039	−.018	.029	.033	.860 *	.745
MDS-33	Family requests withholding information from patient	−.008	.092	−.043	−.010	.838 *	.728
MDS-34	Family insists on treatment viewed as burdensome	−.044	−.009	.004	.039	.854 *	.730
MDS-35	Patient preference overridden by family decision	−.033	.056	.026	−.075	.858 *	.723
MDS-36	Difficulty balancing family wishes and patient welfare	.015	.024	.013	−.013	.832 *	.711
MDS-37	Avoiding end-of-life discussion because of family conflict	.029	−.004	−.013	.012	.777 *	.617
MDS-38	Cultural preference conflict in end-of-life care	.022	−.046	.029	.023	.792 *	.641

Note. Principal Axis Factoring with Promax rotation (κ = 4), n = 298. Primary loadings are marked with an asterisk. All 38 items loaded on their theoretically expected factor, with primary pattern coefficients from .777 to .899. The largest absolute non-primary coefficient in the entire matrix was .111. Communalities (h^2) ranged from .617 to .803 (M = .723). KMO = .955; Bartlett's $\chi^2(703) = 10507.47$, $p < .001$. The five-factor solution accounted for 72.30% of extracted common variance. F1 = Communication and Autonomy Conflicts; F2 = Unprofessional and Futile Care; F3 = Systemic and Economic Constraints; F4 = Working with Incompetent Colleagues; F5 = Conflicts with Family Wishes.

Supplementary Table S3. Factor correlation matrices and eigenvalues

Panel A. Promax factor correlations, intensity EFA (n = 298)

Factor	F1	F2	F3	F4	F5
F1	—	.392	.425	.429	.289
F2	.392	—	.489	.452	.316
F3	.425	.489	—	.384	.253
F4	.429	.452	.384	—	.348
F5	.289	.316	.253	.348	—

Note. Largest inter-factor correlation = .489.

Panel B. Eigenvalues and parallel analysis, intensity subscale

Factor	Observed eigenvalue	95th percentile of random eigenvalues	Decision
1	14.198	1.842	Retained
2	5.950	1.728	Retained
3	4.149	1.649	Retained
4	2.443	1.581	Retained
5	2.076	1.527	Retained
6	0.508	1.475	Not retained
7	0.455	1.429	Not retained
8	0.440	1.383	Not retained

Note. Parallel analysis based on 1,000 random datasets of matching dimensions (n = 298, p = 38). The fifth observed eigenvalue (2.076) exceeded its random counterpart (1.527), whereas the sixth (0.508) fell far below (1.475). Only the first eight factors are shown.

Panel C. Congruence between intensity and frequency solutions

Dimension	Tucker's ϕ	Interpretation
F1 Communication and Autonomy Conflicts	.993	Excellent
F2 Unprofessional and Futile Care	.987	Excellent
F3 Systemic and Economic Constraints	.988	Excellent
F4 Working with Incompetent Colleagues	.976	Excellent
F5 Conflicts with Family Wishes	.995	Excellent
Mean	.988	Excellent

Note. Values ≥ .95 indicate factorial equivalence (Lorenzo-Seva & ten Berge, 2006). The frequency solution yielded KMO = .943, Bartlett's $\chi^2(703) = 7659.67$, $p < .001$, and accounted for 62.26% of extracted common variance.

Supplementary Table S4. Confirmatory factor analysis: standardised loadings and model comparison

Panel A. Standardised loadings, five-factor intensity model (CFA subsample, n = 242)

Item	Abbreviated content	Factor	Standardised λ	R^2
MDS-01	Unable to voice ethical concern because of hierarchy	F1	.858	.736
MDS-02	Physician ignores nursing assessment	F1	.861	.741
MDS-03	Carrying out orders against clinical judgement	F1	.848	.719
MDS-04	Professional judgement not valued in care decisions	F1	.838	.702
MDS-05	Unable to question inappropriate treatment decisions	F1	.842	.709
MDS-06	Being excluded from ethically important discussions	F1	.846	.716
MDS-07	Pressure to follow decisions perceived as unsafe	F1	.816	.666
MDS-08	Difficulty advocating for patient preferences	F1	.855	.731
MDS-09	Limited opportunity to discuss prognosis or goals	F1	.824	.679
MDS-10	Lack of respect for nursing professional role	F1	.799	.638
MDS-11	Continuing treatment perceived as medically futile	F2	.855	.731
MDS-12	Providing aggressive care with little expected benefit	F2	.843	.711
MDS-13	Performing non-beneficial procedures to satisfy others	F2	.848	.719
MDS-14	Continuing life-sustaining treatment despite poor prognosis	F2	.863	.745
MDS-15	Participating in care viewed as prolonging suffering	F2	.878	.771
MDS-16	Assisting with care judged professionally inappropriate	F2	.873	.762
MDS-17	Witnessing unprofessional care practices	F2	.860	.740
MDS-18	Continuing questionable intervention despite ethical concern	F2	.864	.746
MDS-19	Providing suboptimal care because of resource shortage	F3	.868	.753
MDS-20	Patient care limited by economic pressures	F3	.864	.746
MDS-21	Inability to provide needed care because of cost	F3	.843	.711
MDS-22	Pressure to conceal or avoid reporting errors	F3	.848	.719
MDS-23	Institutional constraints limiting ethically appropriate care	F3	.841	.707
MDS-24	Working with incompetent healthcare providers	F4	.867	.752
MDS-25	Colleagues fail to meet patient care needs	F4	.843	.711
MDS-26	Working with clinically unsafe staff	F4	.897	.805
MDS-27	Working with impaired or unreliable staff	F4	.910	.828
MDS-28	Family excludes capable patient from decisions	F5	.869	.755
MDS-29	Family requests aggressive care despite poor prognosis	F5	.835	.697
MDS-30	Family wishes conflict with patient preferences	F5	.840	.706
MDS-31	Family resists discussion of DNR or treatment limits	F5	.878	.771
MDS-32	Religious or cultural objections complicate standard care	F5	.849	.721
MDS-33	Family requests withholding information from patient	F5	.842	.709
MDS-34	Family insists on treatment viewed as burdensome	F5	.846	.716
MDS-35	Patient preference overridden by family decision	F5	.826	.682
MDS-36	Difficulty balancing family wishes and patient welfare	F5	.858	.736
MDS-37	Avoiding end-of-life discussion because of family conflict	F5	.732	.536
MDS-38	Cultural preference conflict in end-of-life care	F5	.741	.549

Note. All loadings $p < .001$, estimated by maximum likelihood (lavaan). Loadings ranged from .732 to .910.

Panel B. Model comparison

Model	χ^2	df	χ^2/df	CFI	TLI	RMSEA [90% CI]	SRMR
Five-factor model	731.00	655	1.12	.991	.990	.022 [.009, .030]	.034
Unidimensional model	5652.00	665	8.50	.415	.381	.176 [.172, .180]	.172
Difference	4921.00	10	—	.576	.609	.154	.138

Note. n = 242. The chi-square difference test was significant, $\Delta\chi^2(10) = 4921.00$, $p < .001$, indicating that the five-factor model fitted substantially better than a unidimensional alternative.

Panel C. Configural invariance across hospital type (N = 540)

Group	n	CFI	TLI	RMSEA [90% CI]	SRMR
Educational hospitals	275	.996	.995	.015 [.000, .025]	.031
Non-educational hospitals	265	.993	.993	.019 [.005, .027]	.033

Note. The five-factor model was estimated separately within each group. All 38 loadings were significant in both groups ($p < .001$) and every item loaded on its intended factor. The mean absolute difference in loadings between groups was .09 (largest .20, MDS-07), and the largest between-group difference in inter-factor correlations was .116. Metric and scalar invariance were not tested; see Sections 3.5 and 4.4.

Supplementary Table S5. Reliability and convergent validity by dimension

Dimension	k	Cronbach's α	CR	AVE	√AVE	Max r with other factors	Fornell–Larcker
F1 Communication and Autonomy Conflicts	10	.958	.960	.704	.839	.456	Satisfied
F2 Unprofessional and Futile Care	8	.955	.958	.741	.861	.482	Satisfied
F3 Systemic and Economic Constraints	5	.941	.930	.728	.853	.474	Satisfied
F4 Working with Incompetent Colleagues	4	.925	.932	.774	.880	.482	Satisfied
F5 Conflicts with Family Wishes	11	.963	.960	.689	.830	.427	Satisfied
Total scale	38	.956	—	—	—	—	—

Note. α computed on the full analytic sample (N = 540); CR and AVE derived from the confirmatory model (n = 242). All AVE values exceed .50 and all CR values exceed .70. The Fornell–Larcker criterion is satisfied for every dimension, since √AVE exceeds the maximum correlation with any other dimension.

Supplementary Table S6. Hierarchical regression predicting burnout and turnover intention (N = 540)

Model	R^2 (EE)	ΔR^2	$F\Delta$	p	R^2 (DP)	ΔR^2	$F\Delta$	p	R^2 (TI)	ΔR^2	$F\Delta$	p
Step 1: covariates	.029	.029	1.96	.050	.005	.005	0.36	.941	.011	.011	0.75	.649
Step 2: + five dimensions	.522	.494	108.77	<.001	.410	.405	72.19	<.001	.477	.466	93.70	<.001

Note. EE = emotional exhaustion; DP = depersonalisation; TI = turnover intention. Step 1 covariates were age, gender, marital status, education, ED experience, employment status, shift type, and hospital type. FΔ df = (8, 531) for Step 1 and (5, 526) for Step 2.

Standardised coefficients (β) from Step 2

Dimension	β (EE)	p	β (DP)	p	β (TI)	p
F1 Communication and Autonomy Conflicts	.341	<.001	.313	<.001	.274	<.001
F2 Unprofessional and Futile Care	.185	<.001	.177	<.001	.211	<.001
F3 Systemic and Economic Constraints	.221	<.001	.212	<.001	.216	<.001
F4 Working with Incompetent Colleagues	.142	<.001	.113	.005	.161	<.001
F5 Conflicts with Family Wishes	.081	.016	.054	.147	.091	.010

Note. All coefficients index concurrent association and do not establish direction of influence. Communication and Autonomy Conflicts showed the strongest unique association with every outcome.
