# Supplementary material for: Dimensionality of Moral Distress Among Emergency Nurses in a Non‐Western Context: A Two‐Stage Psychometric Validation
Source: J Nurs Manag. 2026 Oct 15;2026:1438300. doi: 10.1155/jonm/1438300 (PMC13670431; doi:10.1155/jonm/1438300)
Supplement: Supplementary file 1 [file JONM-2026-1438300-s001.zip › 6_Supplementary_Tables_S7-S8_1438300.docx]

**Supplementary Material — Tables S7 and S8**

*Dimensionality of Moral Distress among Emergency Nurses in a Non-Western Context: A Two-Stage Psychometric Validation (MS 1438300)*

**Table S7.** Clinical interpretation and exemplar item content for the five moral distress dimensions

| **Dimension** | **Clinical meaning** | **Exemplar item content** |
| --- | --- | --- |
| F1 Communication and Autonomy Conflicts | Constrained professional voice; nursing assessment disregarded; orders contrary to clinical judgement | Unable to voice ethical concern; physician disregards nursing assessment; carrying out orders against judgement |
| F2 Unprofessional and Futile Care | Non-beneficial or disproportionate treatment; ethically troubling continuation of care | Continuing treatment perceived as futile; performing non-beneficial procedures; assisting inappropriate practice |
| F3 Systemic and Economic Constraints | Resource shortage; cost-driven limitation of care; pressure surrounding error reporting | Suboptimal care due to resource shortage; care limited by cost; pressure to avoid reporting errors |
| F4 Working with Incompetent Colleagues | Practising alongside incompetent, impaired, or underperforming providers | Working with incompetent providers; colleagues failing to meet patient needs; impaired staff |
| F5 Conflicts with Family Wishes | Family override of patient preference; conflict over treatment limitation; cultural or religious objection | Family insists on aggressive care; patient excluded from decisions; religious objection to standard care |

**Note.** This table presents the interpretive material formerly carried in the main-text Table 4. It was moved to the supplementary material in revision so that Table 4 could serve a single purpose, namely the reporting of numerical descriptive statistics. Exemplar item content is paraphrased; the full item set is available from the corresponding author subject to the terms of the instrument licence.

**Table S8.** Candidate intervention targets mapped to moral distress dimensions

| **Dimension** | **Candidate intervention target** | **Rationale and evidential status** |
| --- | --- | --- |
| F1 Communication and Autonomy Conflicts | Structured interprofessional rounds; SBAR- based escalation protocols; protected channels for raising ethical concern | Strongest cross-sectional association with all three outcomes. Mechanisms addressing constrained voice are plausible but untested here; requires prospective evaluation |
| F2 Unprofessional and Futile Care | Early palliative care triggers; accessible ethics consultation; proactive family conferences | Consistent with reported reductions in distress following ethics consultation services (Hamric & Epstein, 2017); not evaluated in the present sample |
| F3 Systemic and Economic Constraints | Transparent priority-setting; just-culture reporting systems; periodic resource- allocation review | Second strongest associations. Reporting pressure loaded with resource constraint, suggesting shared organisational origin; causal direction untested |
| F4 Working with Incompetent Colleagues | Competency-based remediation pathways; structured peer review; simulation-based training | Smaller but consistent associations. Addresses a distinct constraint class identified by the factor structure |
| F5 Conflicts with Family Wishes | Cultural mediation; access to Islamic bioethics consultation; nurse-led family meetings | Lowest mean intensity, but interpretation is contested (Section 4.5); intervention priority should await clarification of whether low intensity reflects congruence, exposure, or habituation |

**Note.** This table presents hypotheses derived from the dimensional structure and its observed associations with outcomes. It contains no new data and was therefore moved from the main text to the supplementary material in revision. No intervention was implemented or evaluated in this study, and every entry requires prospective testing.
