# Supplementary material for: Dimensionality of Moral Distress Among Emergency Nurses in a Non‐Western Context: A Two‐Stage Psychometric Validation
Source: J Nurs Manag. 2026 Oct 15;2026:1438300. doi: 10.1155/jonm/1438300 (PMC13670431; doi:10.1155/jonm/1438300)
Supplement: Supplementary file 1 [file JONM-2026-1438300-s001.zip › Supplementary_File_A_Permission.pdf]

Supplementary File A

Permission to use the Moral Distress Scale

Journal of Nursing Management — Manuscript ID 1438300

Written permission for use of the 38-item Moral Distress Scale (Corley, Elswick, Gorman & Clor, 2001; Corley, Minick, Elswick & Jacobs, 2005) was granted by the instrument's lead developer, Professor Mary C. Corley, on 30 July 2026. Her email is reproduced on the following page.

Permission granted

> *"You have my permission to use the Moral Distress Scale."*
>
> Professor Mary C. Corley, PhD, RN
> Associate Professor Emerita, Virginia Commonwealth University
> 30 July 2026

Context

Professor Corley retired in 2014, and her published institutional address is no longer monitored. On retirement she transferred responsibility for the instrument to Professor Ann B. Hamric, who died unexpectedly before that responsibility could be passed on, leaving the scale without a clearly identified custodian. Permission was therefore obtained after approaching the surviving co-authors of the original publication, none of whom raised any objection: Professor R. K. Elswick Jr. (Virginia Commonwealth University), second author of Corley et al. (2001), and Professor Beth Epstein (University of Virginia), who oversees the currently distributed revised version. Professor Epstein forwarded the request to Professor Corley, whose reply follows.

Scope

The permission covers use, translation and cultural adaptation of the instrument for the present study. The authors have not reproduced the full item set in the published article. Should the journal wish the translated instrument to be deposited, the authors will provide the Persian and English versions on request.

The permission email is reproduced on the following page.

Permission email

Reproduced verbatim from Gmail

**Subject**	Moral Distress Scale Use
**From**	Mary Corley <mccorley38@gmail.com>
**To**	vzamanzadeh@gmail.com
**Cc**	Beth Epstein <meg4u@virginia.edu>
**Date**	Thursday, 30 July 2026, 03:47

Dear Dr. Zamanzadeh,

You have my permission to use the Moral Distress Scale. Because I am retired, please use my gmail account to communicate with me.

When I retired in 2014, I turned my research over to Dr Ann Hamric, who was to be the contact person for the Moral Distress Scale. She died very unexpectedly and may not have transferred responsibility to Dr. Epstein. I would suggest that you review the research on Moral Distress and Moral Resilience.

Dr. Hamric was very interested in evaluating interventions that would address nurse moral distress.

I wish you well with your research.

Mary Corley

This message was received in response to a permission request submitted through the surviving co-authors of the original instrument. The original email is available from the corresponding author on request.
