# Supplementary material for: Dimensionality of Moral Distress Among Emergency Nurses in a Non‐Western Context: A Two‐Stage Psychometric Validation
Source: J Nurs Manag. 2026 Oct 15;2026:1438300. doi: 10.1155/jonm/1438300 (PMC13670431; doi:10.1155/jonm/1438300)
Supplement: Supplementary file 1 [file JONM-2026-1438300-s001.zip › Supplementary_Tables_S1-S6.docx]

**Supplementary Material**

*Dimensionality of Moral Distress among Emergency Nurses in a Non-Western Context: A Two-Stage Psychometric Validation*

Journal of Nursing Management — Manuscript ID 1438300

**Supplementary Table S1. Item analysis for the Moral Distress Scale intensity subscale (N = 540)**

| **Item** | **Abbreviated content** | **Factor** | **M** | **SD** | **Difficulty** | **Scale M if deleted** | **Scale var. if deleted** | **Corrected item–total r** | **α if deleted** |
| --- | --- | --- | --- | --- | --- | --- | --- | --- | --- |
| MDS-01 | Unable to voice ethical concern because of hierarchy | F1 | 3.57 | 1.86 | 0.59 | 124.09 | 1727.4 | 0.615 | 0.954 |
| MDS-02 | Physician ignores nursing assessment | F1 | 3.53 | 1.85 | 0.59 | 124.14 | 1727.7 | 0.606 | 0.954 |
| MDS-03 | Carrying out orders against clinical judgement | F1 | 3.64 | 1.91 | 0.61 | 124.08 | 1726.7 | 0.607 | 0.954 |
| MDS-04 | Professional judgement not valued in care decisions | F1 | 3.58 | 1.79 | 0.60 | 124.20 | 1726.8 | 0.618 | 0.954 |
| MDS-05 | Unable to question inappropriate treatment decisions | F1 | 3.50 | 1.81 | 0.58 | 124.22 | 1731.0 | 0.593 | 0.955 |
| MDS-06 | Being excluded from ethically important discussions | F1 | 3.47 | 1.84 | 0.58 | 124.24 | 1730.1 | 0.602 | 0.954 |
| MDS-07 | Pressure to follow decisions perceived as unsafe | F1 | 3.33 | 1.79 | 0.56 | 124.26 | 1737.0 | 0.555 | 0.955 |
| MDS-08 | Difficulty advocating for patient preferences | F1 | 3.45 | 1.84 | 0.58 | 124.27 | 1729.5 | 0.602 | 0.954 |
| MDS-09 | Limited opportunity to discuss prognosis or goals | F1 | 3.40 | 1.82 | 0.57 | 124.26 | 1734.6 | 0.572 | 0.955 |
| MDS-10 | Lack of respect for nursing professional role | F1 | 3.23 | 1.73 | 0.54 | 124.45 | 1741.6 | 0.539 | 0.955 |
| MDS-11 | Continuing treatment perceived as medically futile | F2 | 3.51 | 1.81 | 0.58 | 124.24 | 1726.3 | 0.626 | 0.954 |
| MDS-12 | Providing aggressive care with little expected benefit | F2 | 3.62 | 1.83 | 0.60 | 124.29 | 1723.8 | 0.639 | 0.954 |
| MDS-13 | Performing non-beneficial procedures to satisfy others | F2 | 3.56 | 1.78 | 0.59 | 124.27 | 1728.4 | 0.609 | 0.954 |
| MDS-14 | Continuing life-sustaining treatment despite poor prognosis | F2 | 3.51 | 1.85 | 0.59 | 124.28 | 1730.3 | 0.588 | 0.955 |
| MDS-15 | Participating in care viewed as prolonging suffering | F2 | 3.44 | 1.87 | 0.57 | 124.30 | 1725.0 | 0.625 | 0.954 |
| MDS-16 | Assisting with care judged professionally inappropriate | F2 | 3.56 | 1.90 | 0.59 | 124.25 | 1725.6 | 0.609 | 0.954 |
| MDS-17 | Witnessing unprofessional care practices | F2 | 3.46 | 1.78 | 0.58 | 124.36 | 1731.9 | 0.593 | 0.955 |
| MDS-18 | Continuing questionable intervention despite ethical concern | F2 | 3.46 | 1.79 | 0.58 | 124.36 | 1727.0 | 0.636 | 0.954 |
| MDS-19 | Providing suboptimal care because of resource shortage | F3 | 3.31 | 1.88 | 0.55 | 124.36 | 1729.0 | 0.564 | 0.955 |
| MDS-20 | Patient care limited by economic pressures | F3 | 3.21 | 1.97 | 0.54 | 124.43 | 1723.2 | 0.575 | 0.955 |
| MDS-21 | Inability to provide needed care because of cost | F3 | 3.31 | 1.86 | 0.55 | 124.42 | 1731.4 | 0.552 | 0.955 |
| MDS-22 | Pressure to conceal or avoid reporting errors | F3 | 3.18 | 1.83 | 0.53 | 124.45 | 1729.6 | 0.570 | 0.955 |
| MDS-23 | Institutional constraints limiting ethically appropriate care | F3 | 3.19 | 1.87 | 0.53 | 124.51 | 1729.1 | 0.569 | 0.955 |
| MDS-24 | Working with incompetent healthcare providers | F4 | 3.70 | 1.93 | 0.62 | 124.15 | 1733.0 | 0.557 | 0.955 |
| MDS-25 | Colleagues fail to meet patient care needs | F4 | 3.60 | 1.86 | 0.60 | 124.17 | 1737.7 | 0.539 | 0.955 |
| MDS-26 | Working with clinically unsafe staff | F4 | 3.69 | 1.89 | 0.61 | 124.14 | 1731.5 | 0.568 | 0.955 |
| MDS-27 | Working with impaired or unreliable staff | F4 | 3.72 | 1.86 | 0.62 | 124.08 | 1735.3 | 0.555 | 0.955 |
| MDS-28 | Family excludes capable patient from decisions | F5 | 3.52 | 1.88 | 0.59 | 124.41 | 1725.2 | 0.591 | 0.955 |
| MDS-29 | Family requests aggressive care despite poor prognosis | F5 | 3.49 | 1.79 | 0.58 | 124.39 | 1728.5 | 0.592 | 0.955 |
| MDS-30 | Family wishes conflict with patient preferences | F5 | 3.44 | 1.89 | 0.57 | 124.37 | 1723.1 | 0.597 | 0.955 |
| MDS-31 | Family resists discussion of DNR or treatment limits | F5 | 3.50 | 1.81 | 0.58 | 124.45 | 1723.2 | 0.616 | 0.954 |
| MDS-32 | Religious or cultural objections complicate standard care | F5 | 3.34 | 1.88 | 0.56 | 124.51 | 1724.5 | 0.597 | 0.954 |
| MDS-33 | Family requests withholding information from patient | F5 | 3.47 | 1.85 | 0.58 | 124.46 | 1724.8 | 0.603 | 0.954 |
| MDS-34 | Family insists on treatment viewed as burdensome | F5 | 3.41 | 1.81 | 0.57 | 124.43 | 1728.2 | 0.586 | 0.955 |
| MDS-35 | Patient preference overridden by family decision | F5 | 3.37 | 1.80 | 0.56 | 124.51 | 1729.9 | 0.582 | 0.955 |
| MDS-36 | Difficulty balancing family wishes and patient welfare | F5 | 3.21 | 1.81 | 0.54 | 124.56 | 1725.9 | 0.621 | 0.954 |
| MDS-37 | Avoiding end-of-life discussion because of family conflict | F5 | 2.75 | 1.60 | 0.46 | 125.03 | 1744.5 | 0.549 | 0.955 |
| MDS-38 | Cultural preference conflict in end-of-life care | F5 | 2.74 | 1.62 | 0.46 | 125.07 | 1746.5 | 0.540 | 0.955 |

*Note. N = 540. Difficulty index = item mean / maximum possible score (6). Corrected item–total correlations ranged from .539 to .639 (M = .588), all exceeding the .30 criterion and indeed the more stringent .50 threshold. Total-scale Cronbach’s α = .956; no item deletion would have increased it. Items MDS-37 and MDS-38, identified during cognitive interviewing as potentially double-barrelled, showed the lowest item means (2.75) but acceptable item–total correlations (.549 and .540) and were retained.*

**Supplementary Table S2. Complete rotated pattern matrix, intensity subscale (EFA subsample, n = 298)**

| **Item** | **Abbreviated content** | **F1** | **F2** | **F3** | **F4** | **F5** | **h²** |
| --- | --- | --- | --- | --- | --- | --- | --- |
| MDS-01 | Unable to voice ethical concern because of hierarchy | .859 * | −.035 | .016 | .007 | .009 | .738 |
| MDS-02 | Physician ignores nursing assessment | .847 * | −.044 | .076 | −.056 | .059 | .739 |
| MDS-03 | Carrying out orders against clinical judgement | .863 * | .018 | .016 | −.036 | .013 | .749 |
| MDS-04 | Professional judgement not valued in care decisions | .786 * | .098 | −.055 | −.005 | .053 | .674 |
| MDS-05 | Unable to question inappropriate treatment decisions | .837 * | .037 | −.002 | −.025 | .032 | .723 |
| MDS-06 | Being excluded from ethically important discussions | .819 * | −.043 | .036 | −.026 | .074 | .691 |
| MDS-07 | Pressure to follow decisions perceived as unsafe | .815 * | −.024 | −.030 | .078 | −.102 | .647 |
| MDS-08 | Difficulty advocating for patient preferences | .847 * | −.019 | −.013 | .075 | −.099 | .712 |
| MDS-09 | Limited opportunity to discuss prognosis or goals | .801 * | .023 | −.014 | .018 | −.020 | .650 |
| MDS-10 | Lack of respect for nursing professional role | .804 * | .000 | −.008 | −.031 | −.007 | .618 |
| MDS-11 | Continuing treatment perceived as medically futile | .006 | .851 * | .021 | .008 | −.001 | .753 |
| MDS-12 | Providing aggressive care with little expected benefit | .051 | .795 * | −.010 | .016 | .067 | .712 |
| MDS-13 | Performing non-beneficial procedures to satisfy others | .027 | .861 * | −.025 | −.018 | −.009 | .721 |
| MDS-14 | Continuing life-sustaining treatment despite poor prognosis | −.111 | .877 * | .043 | −.050 | .016 | .714 |
| MDS-15 | Participating in care viewed as prolonging suffering | .043 | .803 * | −.042 | .036 | .022 | .681 |
| MDS-16 | Assisting with care judged professionally inappropriate | .012 | .793 * | .064 | .028 | −.041 | .692 |
| MDS-17 | Witnessing unprofessional care practices | −.050 | .846 * | .071 | −.045 | .005 | .716 |
| MDS-18 | Continuing questionable intervention despite ethical concern | .043 | .885 * | −.083 | .050 | −.057 | .758 |
| MDS-19 | Providing suboptimal care because of resource shortage | −.045 | .055 | .867 * | −.027 | .020 | .760 |
| MDS-20 | Patient care limited by economic pressures | .042 | .007 | .866 * | .017 | −.028 | .788 |
| MDS-21 | Inability to provide needed care because of cost | .019 | −.027 | .876 * | .025 | −.013 | .770 |
| MDS-22 | Pressure to conceal or avoid reporting errors | −.011 | .004 | .897 * | .012 | −.009 | .803 |
| MDS-23 | Institutional constraints limiting ethically appropriate care | .028 | .010 | .872 * | −.007 | .022 | .796 |
| MDS-24 | Working with incompetent healthcare providers | .031 | .030 | −.009 | .834 * | −.009 | .731 |
| MDS-25 | Colleagues fail to meet patient care needs | −.037 | .018 | .032 | .867 * | −.012 | .754 |
| MDS-26 | Working with clinically unsafe staff | .012 | −.009 | −.028 | .860 * | .039 | .747 |
| MDS-27 | Working with impaired or unreliable staff | .013 | −.012 | .028 | .822 * | .023 | .708 |
| MDS-28 | Family excludes capable patient from decisions | .009 | −.073 | −.015 | .011 | .899 * | .777 |
| MDS-29 | Family requests aggressive care despite poor prognosis | .088 | .007 | −.022 | −.080 | .852 * | .724 |
| MDS-30 | Family wishes conflict with patient preferences | −.039 | −.057 | .008 | .043 | .898 * | .789 |
| MDS-31 | Family resists discussion of DNR or treatment limits | −.005 | .032 | −.027 | .052 | .839 * | .741 |
| MDS-32 | Religious or cultural objections complicate standard care | −.039 | −.018 | .029 | .033 | .860 * | .745 |
| MDS-33 | Family requests withholding information from patient | −.008 | .092 | −.043 | −.010 | .838 * | .728 |
| MDS-34 | Family insists on treatment viewed as burdensome | −.044 | −.009 | .004 | .039 | .854 * | .730 |
| MDS-35 | Patient preference overridden by family decision | −.033 | .056 | .026 | −.075 | .858 * | .723 |
| MDS-36 | Difficulty balancing family wishes and patient welfare | .015 | .024 | .013 | −.013 | .832 * | .711 |
| MDS-37 | Avoiding end-of-life discussion because of family conflict | .029 | −.004 | −.013 | .012 | .777 * | .617 |
| MDS-38 | Cultural preference conflict in end-of-life care | .022 | −.046 | .029 | .023 | .792 * | .641 |

*Note. Principal Axis Factoring with Promax rotation (κ = 4), n = 298. Primary loadings are marked with an asterisk. All 38 items loaded on their theoretically expected factor, with primary pattern coefficients from .777 to .899. The largest absolute non-primary coefficient in the entire matrix was .111. Communalities (h²) ranged from .617 to .803 (M = .723). KMO = .955; Bartlett’s χ²(703) = 10507.47, p < .001. The five-factor solution accounted for 72.30% of extracted common variance. F1 = Communication and Autonomy Conflicts; F2 = Unprofessional and Futile Care; F3 = Systemic and Economic Constraints; F4 = Working with Incompetent Colleagues; F5 = Conflicts with Family Wishes.*

**Supplementary Table S3. Factor correlation matrices and eigenvalues**

**Panel A. Promax factor correlations, intensity EFA (n = 298)**

| **Factor** | **F1** | **F2** | **F3** | **F4** | **F5** |
| --- | --- | --- | --- | --- | --- |
| F1 | — | .392 | .425 | .429 | .289 |
| F2 | .392 | — | .489 | .452 | .316 |
| F3 | .425 | .489 | — | .384 | .253 |
| F4 | .429 | .452 | .384 | — | .348 |
| F5 | .289 | .316 | .253 | .348 | — |

*Note. Largest inter-factor correlation = .489.*

**Panel B. Eigenvalues and parallel analysis, intensity subscale**

| **Factor** | **Observed eigenvalue** | **95th percentile of random eigenvalues** | **Decision** |
| --- | --- | --- | --- |
| 1 | 14.198 | 1.842 | Retained |
| 2 | 5.950 | 1.728 | Retained |
| 3 | 4.149 | 1.649 | Retained |
| 4 | 2.443 | 1.581 | Retained |
| 5 | 2.076 | 1.527 | Retained |
| 6 | 0.508 | 1.475 | Not retained |
| 7 | 0.455 | 1.429 | Not retained |
| 8 | 0.440 | 1.383 | Not retained |

*Note. Parallel analysis based on 1,000 random datasets of matching dimensions (n = 298, p = 38). The fifth observed eigenvalue (2.076) exceeded its random counterpart (1.527), whereas the sixth (0.508) fell far below (1.475). Only the first eight factors are shown.*

**Panel C. Congruence between intensity and frequency solutions**

| **Dimension** | **Tucker’s φ** | **Interpretation** |
| --- | --- | --- |
| F1 Communication and Autonomy Conflicts | .993 | Excellent |
| F2 Unprofessional and Futile Care | .987 | Excellent |
| F3 Systemic and Economic Constraints | .988 | Excellent |
| F4 Working with Incompetent Colleagues | .976 | Excellent |
| F5 Conflicts with Family Wishes | .995 | Excellent |
| Mean | .988 | Excellent |

*Note. Values ≥ .95 indicate factorial equivalence (Lorenzo-Seva & ten Berge, 2006). The frequency solution yielded KMO = .943, Bartlett’s χ²(703) = 7659.67, p < .001, and accounted for 62.26% of extracted common variance.*

**Supplementary Table S4. Confirmatory factor analysis: standardised loadings and model comparison**

**Panel A. Standardised loadings, five-factor intensity model (CFA subsample, n = 242)**

| **Item** | **Abbreviated content** | **Factor** | **Standardised λ** | **R²** |
| --- | --- | --- | --- | --- |
| MDS-01 | Unable to voice ethical concern because of hierarchy | F1 | .858 | .736 |
| MDS-02 | Physician ignores nursing assessment | F1 | .861 | .741 |
| MDS-03 | Carrying out orders against clinical judgement | F1 | .848 | .719 |
| MDS-04 | Professional judgement not valued in care decisions | F1 | .838 | .702 |
| MDS-05 | Unable to question inappropriate treatment decisions | F1 | .842 | .709 |
| MDS-06 | Being excluded from ethically important discussions | F1 | .846 | .716 |
| MDS-07 | Pressure to follow decisions perceived as unsafe | F1 | .816 | .666 |
| MDS-08 | Difficulty advocating for patient preferences | F1 | .855 | .731 |
| MDS-09 | Limited opportunity to discuss prognosis or goals | F1 | .824 | .679 |
| MDS-10 | Lack of respect for nursing professional role | F1 | .799 | .638 |
| MDS-11 | Continuing treatment perceived as medically futile | F2 | .855 | .731 |
| MDS-12 | Providing aggressive care with little expected benefit | F2 | .843 | .711 |
| MDS-13 | Performing non-beneficial procedures to satisfy others | F2 | .848 | .719 |
| MDS-14 | Continuing life-sustaining treatment despite poor prognosis | F2 | .863 | .745 |
| MDS-15 | Participating in care viewed as prolonging suffering | F2 | .878 | .771 |
| MDS-16 | Assisting with care judged professionally inappropriate | F2 | .873 | .762 |
| MDS-17 | Witnessing unprofessional care practices | F2 | .860 | .740 |
| MDS-18 | Continuing questionable intervention despite ethical concern | F2 | .864 | .746 |
| MDS-19 | Providing suboptimal care because of resource shortage | F3 | .868 | .753 |
| MDS-20 | Patient care limited by economic pressures | F3 | .864 | .746 |
| MDS-21 | Inability to provide needed care because of cost | F3 | .843 | .711 |
| MDS-22 | Pressure to conceal or avoid reporting errors | F3 | .848 | .719 |
| MDS-23 | Institutional constraints limiting ethically appropriate care | F3 | .841 | .707 |
| MDS-24 | Working with incompetent healthcare providers | F4 | .867 | .752 |
| MDS-25 | Colleagues fail to meet patient care needs | F4 | .843 | .711 |
| MDS-26 | Working with clinically unsafe staff | F4 | .897 | .805 |
| MDS-27 | Working with impaired or unreliable staff | F4 | .910 | .828 |
| MDS-28 | Family excludes capable patient from decisions | F5 | .869 | .755 |
| MDS-29 | Family requests aggressive care despite poor prognosis | F5 | .835 | .697 |
| MDS-30 | Family wishes conflict with patient preferences | F5 | .840 | .706 |
| MDS-31 | Family resists discussion of DNR or treatment limits | F5 | .878 | .771 |
| MDS-32 | Religious or cultural objections complicate standard care | F5 | .849 | .721 |
| MDS-33 | Family requests withholding information from patient | F5 | .842 | .709 |
| MDS-34 | Family insists on treatment viewed as burdensome | F5 | .846 | .716 |
| MDS-35 | Patient preference overridden by family decision | F5 | .826 | .682 |
| MDS-36 | Difficulty balancing family wishes and patient welfare | F5 | .858 | .736 |
| MDS-37 | Avoiding end-of-life discussion because of family conflict | F5 | .732 | .536 |
| MDS-38 | Cultural preference conflict in end-of-life care | F5 | .741 | .549 |

*Note. All loadings p < .001, estimated by maximum likelihood (lavaan). Loadings ranged from .732 to .910.*

**Panel B. Model comparison**

| **Model** | **χ²** | **df** | **χ²/df** | **CFI** | **TLI** | **RMSEA [90% CI]** | **SRMR** |
| --- | --- | --- | --- | --- | --- | --- | --- |
| Five-factor model | 731.00 | 655 | 1.12 | .991 | .990 | .022 [.009, .030] | .034 |
| Unidimensional model | 5652.00 | 665 | 8.50 | .415 | .381 | .176 [.172, .180] | .172 |
| Difference | 4921.00 | 10 | — | .576 | .609 | .154 | .138 |

*Note. n = 242. The chi-square difference test was significant, Δχ²(10) = 4921.00, p < .001, indicating that the five-factor model fitted substantially better than a unidimensional alternative.*

**Panel C. Configural invariance across hospital type (N = 540)**

| **Group** | **n** | **CFI** | **TLI** | **RMSEA [90% CI]** | **SRMR** |
| --- | --- | --- | --- | --- | --- |
| Educational hospitals | 275 | .996 | .995 | .015 [.000, .025] | .031 |
| Non-educational hospitals | 265 | .993 | .993 | .019 [.005, .027] | .033 |

*Note. The five-factor model was estimated separately within each group. All 38 loadings were significant in both groups (p < .001) and every item loaded on its intended factor. The mean absolute difference in loadings between groups was .09 (largest .20, MDS-07), and the largest between-group difference in inter-factor correlations was .116. Metric and scalar invariance were not tested; see Sections 3.5 and 4.4.*

**Supplementary Table S5. Reliability and convergent validity by dimension**

| **Dimension** | **k** | **Cronbach’s α** | **CR** | **AVE** | **√AVE** | **Max r with other factors** | **Fornell–Larcker** |
| --- | --- | --- | --- | --- | --- | --- | --- |
| F1 Communication and Autonomy Conflicts | 10 | .958 | .960 | .704 | .839 | .456 | Satisfied |
| F2 Unprofessional and Futile Care | 8 | .955 | .958 | .741 | .861 | .482 | Satisfied |
| F3 Systemic and Economic Constraints | 5 | .941 | .930 | .728 | .853 | .474 | Satisfied |
| F4 Working with Incompetent Colleagues | 4 | .925 | .932 | .774 | .880 | .482 | Satisfied |
| F5 Conflicts with Family Wishes | 11 | .963 | .960 | .689 | .830 | .427 | Satisfied |
| Total scale | 38 | .956 | — | — | — | — | — |

*Note. α computed on the full analytic sample (N = 540); CR and AVE derived from the confirmatory model (n = 242). All AVE values exceed .50 and all CR values exceed .70. The Fornell–Larcker criterion is satisfied for every dimension, since √AVE exceeds the maximum correlation with any other dimension.*

**Supplementary Table S6. Hierarchical regression predicting burnout and turnover intention (N = 540)**

| **Model** | **R² (EE)** | **ΔR²** | **FΔ** | **p** | **R² (DP)** | **ΔR²** | **FΔ** | **p** | **R² (TI)** | **ΔR²** | **FΔ** | **p** |
| --- | --- | --- | --- | --- | --- | --- | --- | --- | --- | --- | --- | --- |
| Step 1: covariates | .029 | .029 | 1.96 | .050 | .005 | .005 | 0.36 | .941 | .011 | .011 | 0.75 | .649 |
| Step 2: + five dimensions | .522 | .494 | 108.77 | <.001 | .410 | .405 | 72.19 | <.001 | .477 | .466 | 93.70 | <.001 |

*Note. EE = emotional exhaustion; DP = depersonalisation; TI = turnover intention. Step 1 covariates were age, gender, marital status, education, ED experience, employment status, shift type, and hospital type. FΔ df = (8, 531) for Step 1 and (5, 526) for Step 2.*

**Standardised coefficients (β) from Step 2**

| **Dimension** | **β (EE)** | **p** | **β (DP)** | **p** | **β (TI)** | **p** |
| --- | --- | --- | --- | --- | --- | --- |
| F1 Communication and Autonomy Conflicts | .341 | <.001 | .313 | <.001 | .274 | <.001 |
| F2 Unprofessional and Futile Care | .185 | <.001 | .177 | <.001 | .211 | <.001 |
| F3 Systemic and Economic Constraints | .221 | <.001 | .212 | <.001 | .216 | <.001 |
| F4 Working with Incompetent Colleagues | .142 | <.001 | .113 | .005 | .161 | <.001 |
| F5 Conflicts with Family Wishes | .081 | .016 | .054 | .147 | .091 | .010 |

*Note. All coefficients index concurrent association and do not establish direction of influence. Communication and Autonomy Conflicts showed the strongest unique association with every outcome.*
